# Temperature Modulates the Effects of Ocean Acidification on Intestinal Ion Transport in Atlantic Cod, *Gadus morhua*

**DOI:** 10.3389/fphys.2016.00198

**Published:** 2016-06-02

**Authors:** Marian Y. Hu, Katharina Michael, Cornelia M. Kreiss, Meike Stumpp, Sam Dupont, Yung-Che Tseng, Magnus Lucassen

**Affiliations:** ^1^Institute of Physiology, University of KielKiel, Germany; ^2^Helmholtz Center for Polar and Marine Research, Alfred Wegener InstituteBremerhaven, Germany; ^3^Helmholtz Centre for Ocean Research KielKiel, Germany; ^4^Department of Biological and Environmental Sciences, The Sven Lovén Centre for Marine Sciences, University of GothenburgGothenburg, Sweden; ^5^Department of Life Science, National Taiwan Normal UniversityTaipei City, Taiwan

**Keywords:** thermal compensation, hypercapnia, pH regulation, bicarbonate level, teleost

## Abstract

CO_2_-driven seawater acidification has been demonstrated to enhance intestinal bicarbonate secretion rates in teleosts, leading to an increased release of CaCO_3_ under simulated ocean acidification scenarios. In this study, we investigated if increasing CO_2_ levels stimulate the intestinal acid–base regulatory machinery of Atlantic cod (*Gadus morhua*) and whether temperatures at the upper limit of thermal tolerance stimulate or counteract ion regulatory capacities. Juvenile *G. morhua* were acclimated for 4 weeks to three CO_2_ levels (550, 1200, and 2200 μatm) covering present and near-future natural variability, at optimum (10°C) and summer maximum temperature (18°C), respectively. Immunohistochemical analyses revealed the subcellular localization of ion transporters, including Na^+^/K^+^-ATPase (NKA), Na^+^/H^+^-exchanger 3 (NHE3), Na^+^/${\text{HCO}}_{3}^{-}$ cotransporter (NBC1), pendrin-like Cl^−^/${\text{HCO}}_{3}^{-}$ exchanger (SLC26a6), V-type H^+^-ATPase subunit a (VHA), and Cl^−^ channel 3 (CLC3) in epithelial cells of the anterior intestine. At 10°C, proteins and mRNA were generally up-regulated for most transporters in the intestinal epithelium after acclimation to higher CO_2_ levels. This supports recent findings demonstrating increased intestinal ${\text{HCO}}_{3}^{-}$ secretion rates in response to CO_2_ induced seawater acidification. At 18°C, mRNA expression and protein concentrations of most ion transporters remained unchanged or were even decreased, suggesting thermal compensation. This response may be energetically favorable to retain blood ${\text{HCO}}_{3}^{-}$ levels to stabilize pH_e,_ but may negatively affect intestinal salt and water resorption of marine teleosts in future oceans.

## Introduction

Fish are well known as strong acid-base regulators that are capable of accumulating ${\text{HCO}}_{3}^{-}$ in body fluids to fully compensate for CO_2_ induced acid-base disturbances (Heisler, 1984). Therefore, marine teleosts have been hypothesized to be relatively tolerant towards ocean acidification. However, recent studies demonstrated that CO_2_ induced seawater acidification may negatively affect physiological processes which can be related to acid-base regulation. Nilsson et al. (2012) suggested, that chronic alterations in intra- and extracellular [${\text{HCO}}_{3}^{-}$] and [Cl^−^] affect GABA-A receptor functionality (Nilsson et al., 2012), evoking pathological behavioral defects such as impaired olfactory discrimination, loss of behavioral lateralization and disturbed auditory preferences that may impact survival and fitness of several marine teleost species (Munday et al., 2009; Dixson et al., 2010; Simpson et al., 2011; Nilsson et al., 2012). Furthermore, increased blood ${\text{HCO}}_{3}^{-}$ levels were shown to stimulate intestinal anion exchange (${\text{HCO}}_{3}^{-}$ secretion and Cl^−^ resorption) activity, leading to a seemingly counterproductive loss in ${\text{HCO}}_{3}^{-}$ during exposure to hypercapnic conditions that is probably compensated through ${\text{HCO}}_{3}^{-}$ uptake via branchial epithelia (Heuer et al., 2012).

The marine teleost intestine plays a major role in water balance by using Na^+^ and Cl^−^ to create an osmotic driving force to absorb water from the luminal space (Grosell, 2006; Grosell and Genz, 2006). This process involves ion transporters including the Na^+^/Cl^−^ symporter (NCC) and Na^+^/K^+^/Cl^−^ co-transporter 2 (NKCC2) located in apical membranes, which import Na^+^ and Cl^−^ ions. Ion transport against adverse gradients is fueled by the electrochemical gradient created by the Na^+^/K^+^-ATPase (NKA) located in basolateral membranes of the intestinal epithelium (Grosell and Genz, 2006). Anion exchange through pendrin-like transporter (SLC26a6) contributes to apical Cl^−^ absorption as well (Kurita et al., 2008). Besides transporters, which are directly linked to osmotic homeostasis (e.g., NCC, NKCC2), a range of primary and secondary active transporters were identified in the marine teleost intestine, which also participate in acid-base equivalent exchange, including apical V-type H^+^-ATPases (VHA), anion exchangers (e.g., SLC26a6) and basolateral Na^+^/${\text{HCO}}_{3}^{-}$ exchangers (NBCa). Apical H^+^ secretion via VHA and Na^+^/H^+^ exchangers (NHE), respectively were hypothesized to contribute to H^+^ export and mask parts of the secreted ${\text{HCO}}_{3}^{-}$ by shifting the equilibrium toward H_2_O and CO_2_. This molecular CO_2_ can diffuse back into the cell for re-hydration and repeated apical anion exchange (Grosell, 2011).

In addition to the accumulation of ${\text{HCO}}_{3}^{-}$ in body fluids upon elevated seawater *p*CO_2_, intestinal anion exchange activity was found stimulated, leading to increased ${\text{HCO}}_{3}^{-}$ secretion and CaCO_3_ precipitation in the intestine of teleosts (Heuer et al., 2012). Besides elevated *p*CO_2_ levels, also increased water temperatures can directly affect ion and acid-base regulatory processes in ectothermic animals. For example, long-term thermal acclimation has been demonstrated to affect branchial Na^+^/K^+^-ATPase activity and expression in various teleost species (Staurnes, 1993; Imsland et al., 2003; Metz et al., 2003; Michael et al., 2016a). In the marine teleost intestine, altered transport kinetics of intestinal ${\text{HCO}}_{3}^{-}$ upon changing environmental temperatures were observed. Here, a 10°C decrease in temperature resulted in a 1.8- to 3.0-fold reduction in luminal ${\text{HCO}}_{3}^{-}$ secretion in isolated intestine epithelia of gulf toadfish (*Opsanus beta*) (Grosell and Genz, 2006). Here, a proper metabolic shift fueled through alternative energy sources (e.g., lactate) under hypothermic stress has been demonstrated to be beneficial to counteract activity losses, thus maintaining intact homeostasis in zebrafish (Tseng et al., 2014). To date, combined effects of temperature and CO_2_ on the intestinal pH regulatory machinery, are still poorly investigated for marine teleosts including Atlantic cod, *Gadus morhua*.

This species, found in temperate to polar marine habitats, is of high ecological and commercial importance. *G. morhua* has a demersal lifestyle and mainly feeds on fish as well as benthic and infaunal invertebrates (e.g., crustaceans, annelids etc.; Link and Garrison, 2002). The entire thermal niche determined for 8 different stocks in the northeast Atlantic ranged from 1.5 to 19°C (Righton et al., 2010). 10°C has been shown to be the optimum temperature whereas 18°C is close to the upper thermal limit of the distribution area of this species (Pörtner et al., 2001; Lannig et al., 2004). In this study a population of Atlantic cod from the Skagerrak/Kattegat (Gullmarsfjord, Sweden) was used and animals were exposed to different *p*CO_2_ levels to cover present and future natural variability at the study site: (i) 550 μatm as the average *p*CO_2_ at the study site at 10°C; (ii) 1200 μatm as the present extreme *p*CO_2_ at the study site at 18°C and model projection for the average *p*CO_2_ at 10°C within the next 100 years and, (iii) 2200 μatm at both temperatures in accordance with model predictions for the next 100 years (Dorey et al., 2013; Gräns et al., 2014). We studied the effects of CO_2_ and temperature on the ion regulatory machinery of the anterior intestine of Atlantic cod as this segment can be characterized by highest ${\text{HCO}}_{3}^{-}$ secretion rates compared to other segments (Grosell and Jensen, 1999). Intestinal protein levels for various ion transporters including NKA, VHA, NHE3, NBC1, SLC26a6 and the chloride channel 3 (CLC3) were determined in intestinal tissues after temperature and CO_2_ acclimation. Using the cod genome (http://www.codgenome.no/) and the Ensembl genome browser system (http://www.ensembl.org/), we selected expression sequence information of 29 acid-base transporters and their mRNA expression levels were assessed using quantitative Real-Time PCR (qRT-PCR). Finally, functional capacities of NKA were determined, as this enzyme is believed to provide the major driving force for most energy-dependent ion transport in the fish intestine. We hypothesized that in response to elevated *p*CO_2_ levels (hypercapnia) at 10°C, selected transporters of the intestinal acid-base regulatory machinery will increase expression levels to support an increased secretion of ${\text{HCO}}_{3}^{-}$ into the intestinal lumen. However, at 18°C the reduced aerobic scope of this species may cause energetic limitations leading to a less pronounced stimulation of the intestinal ion regulatory machinery evoked by acidified conditions.

## Results

### Immunohistochemical localization of acid-base transporters

**Figure 1:**
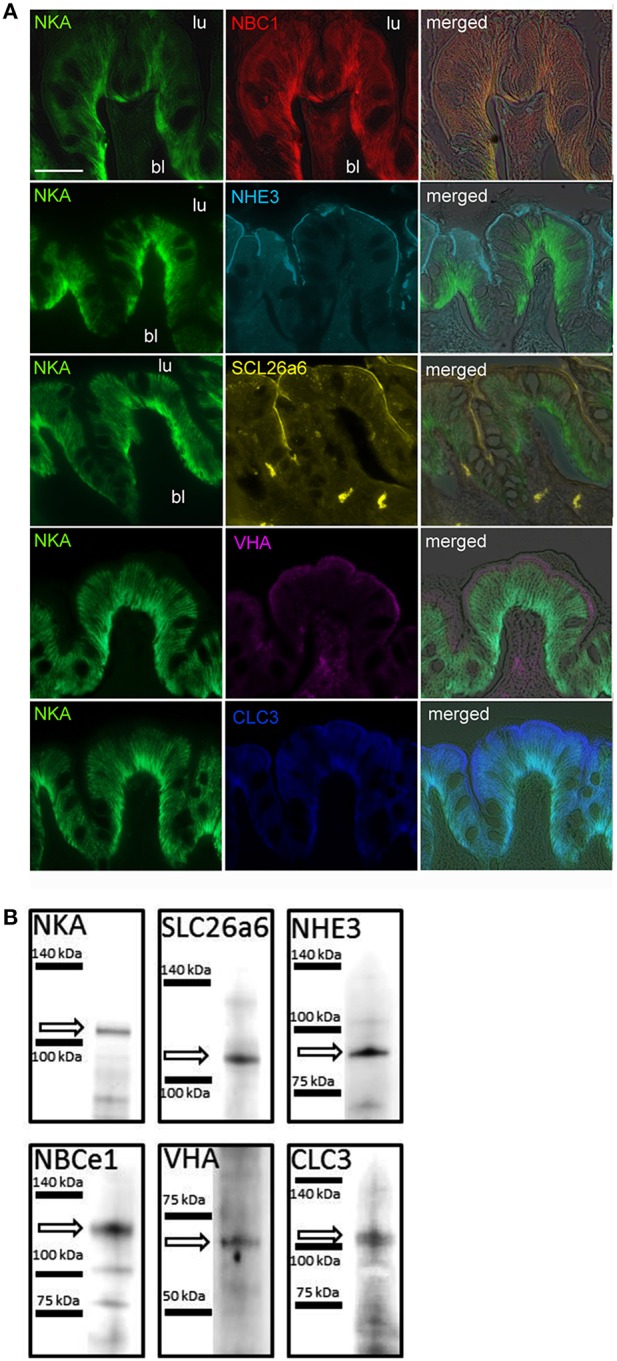
**Localization of acid-base relevant transporters in the anterior intestine of Atlantic cod ***Gadus morhua***. (A)** Transporters including, NBC1 (Na^+^/${\text{HCO}}_{3}^{-}$ -cotransporter1), NKA (Na^+^/K^+^-ATPase), and CLC3 (chloride channel 3) were localized in deep infoldings of basolateral membranes, whereas NHE3 (Na^+^/H^+^-exchanger 3), pendrin-like SLC26a6 (Cl^−^/${\text{HCO}}_{3}^{-}$-exchanger), VHA (V-type H^+^-ATPase), and CLC (chloride channel 3) were localized in apical membranes of intestine epithelial cells. Scale bar 20 μm. **(B)** Western blot analyses using homogenates from anterior intestine tissues demonstrate the specificity of each antibody.

**Figure 2:**
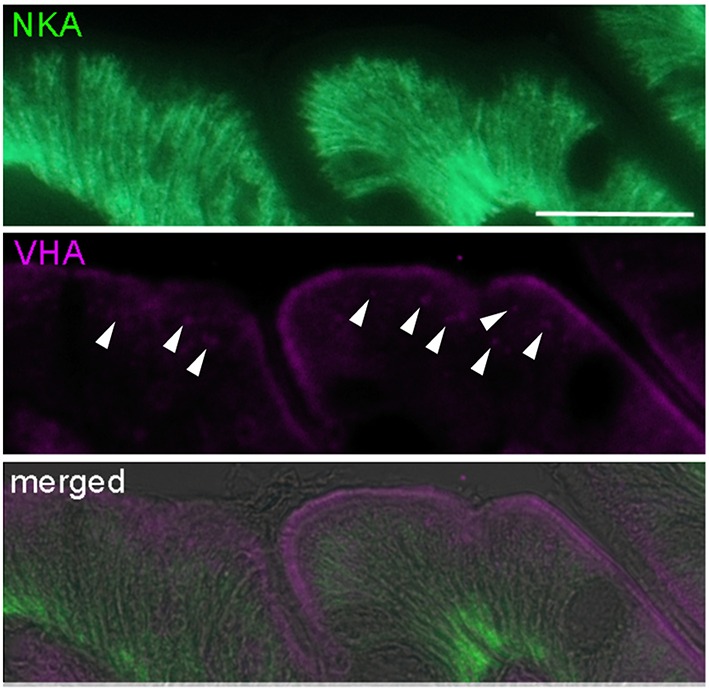
**High magnification image of NKA (green) and VHA (purple) co-localization demonstrating apical localization in the brush border microvilli and sub-apical localization of the VHA**. NKA is localized in basolateral and VHA in apical membranes. Note the fluorescence signal in sub-apical vesicles (indicated by arrow heads). Scale bar 20 μm.

Immunohistological analyses demonstrated that various acid-base relevant transporters including NKA, NHE3, NBC1, SLC26a6, VHA, and CLC3 are localized in apical (luminal) and basolateral membranes of epithelial cells of the cod anterior intestine (Figure 1A). These cells are characterized by deep infoldings of the basolateral membrane (indicated by NKA immunoreactivity; green), almost reaching the apical membrane. Epithelial cells are approximately 20 μm in height and nuclei are visible as dark spots. Using double immunofluorescent staining we could demonstrate positive NKA as well as NBC1 immunoreactivity in basolateral membranes (Figure 1A). Furthermore sub-cellular co-localizations show apical immunoreactivity of the NHE3, pendrin (SLC26a6), and VHA antibodies (Figure 1A). Positive immunoreactivity of NHE3 and pendrin can be characterized by a sharp lining of the apical membrane. However, VHA immunoreactivity appears as a slightly wider lining in apical membranes. Western blot analyses demonstrate specific immunoreactivity with proteins in the predicted size range (Figure 1B). Weak signals of secondary low molecular weight immune-reactive bands were observed for the NKA and NBCe1 antibodies which may be due to slight degradation of the protein leading to breakdown products of the full length protein or due to cross reaction with different isoforms since the NKA antibody was not specifically designed against homologs in cod. High magnification images of NKA and VHA double staining suggest that besides immunoreactivity within the apical membrane, sub-apical vesicles within the cell are positively labeled as well (Figure 2). The polyclonal CLC3 antibody indicates positive immunoreactivity in both basolateral and apical membranes.

### NKA enzyme activity

**Figure 3:**
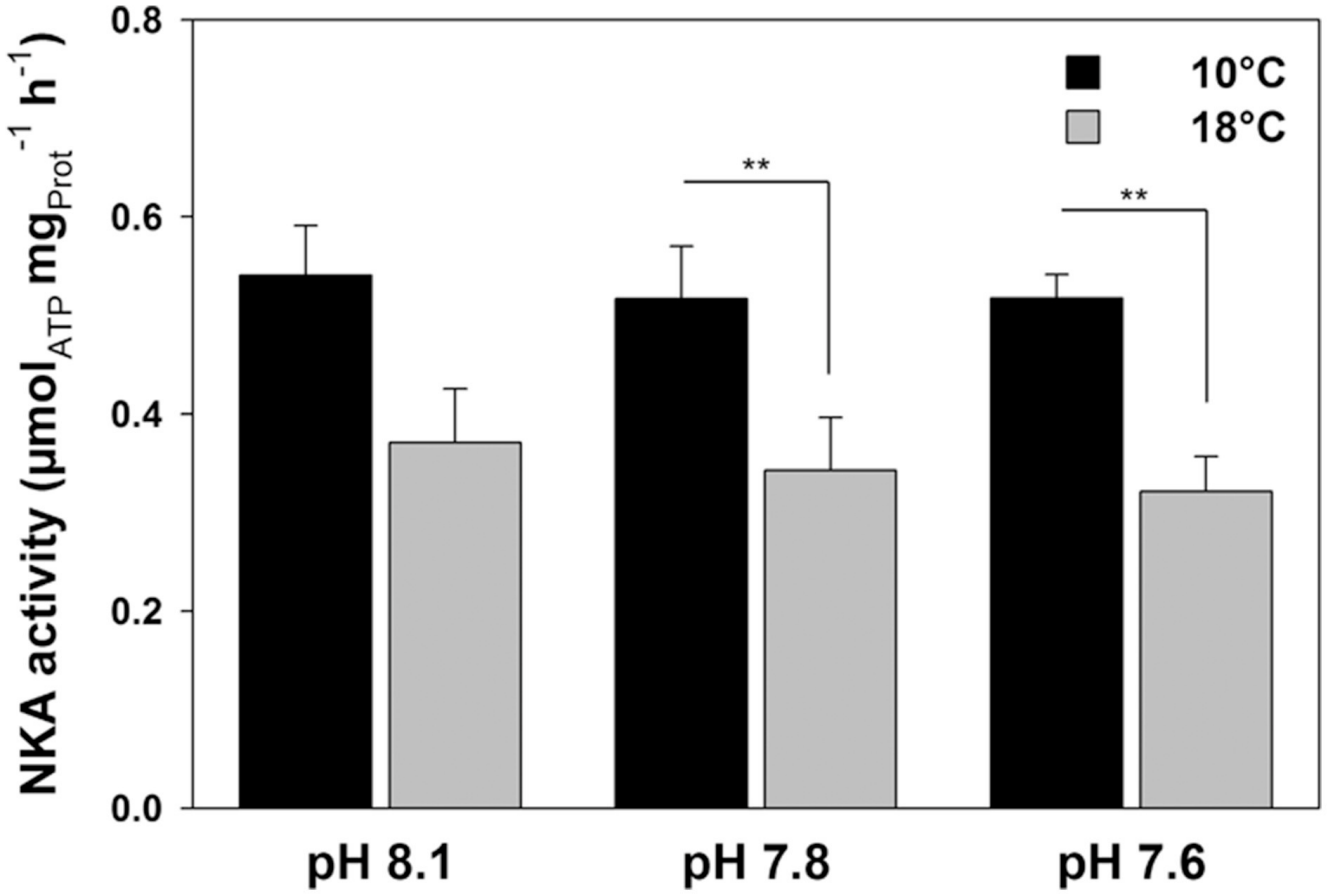
**Determination of maximum NKA (Na^**+**^/K^**+**^-ATPase) activities at 25°C in homogenates of anterior intestine tissues of fish acclimated to three different seawater pH and two temperature levels for 4 weeks**. Asterisks indicate differences between temperature treatments (^**^p < 0.01). Values are given as mean ± SE (*n* = 6–8).

Determination of NKA enzyme activities demonstrated no significant effects of *p*CO_2_, whereas temperature had large effects [Figure 3; two-way ANOVA *p*CO_2_, *df*_(2, 39)_ *F* = 0.319 *p* = 0.729; temp, *df*_(1, 39)_ *F* = 21.064, *p* < 0.001). At 10°C, NKA activities were 0.54 ± 0.05 μmol_ATP_ ${\text{mg}}_{Prot}^{-1}$ h^−1^ (low *p*CO_2_), 0.52 ± 0.05 (intermediate *p*CO_2_) and 0.52 ± 0.02 μmol_ATP_ ${\text{mg}}_{Prot}^{-1}$ h^−1^ (high *p*CO_2_). At 18°C, NKA activities were significantly decreased by about 35–40% (0.37 ± 0.05 μmol_ATP_ ${\text{mg}}_{Prot}^{-1}$ h^−1^ (low *p*CO_2_), 0.34 ± 0.05 (intermediate *p*CO_2_) and 0.32 ± 0.04 μmol_ATP_ ${\text{mg}}_{Prot}^{-1}$ h^−1^ (high *p*CO_2_)] compared to those acclimated to 10°C, when measured at a common temperature of 25°C. No interaction between temperature and CO_2_ has been found (*p* = 0.957). The patterns of NKA capacities related to fresh weight were similar (data not shown).

### Protein concentrations of intestinal acid-base transporters

**Figure 4:**
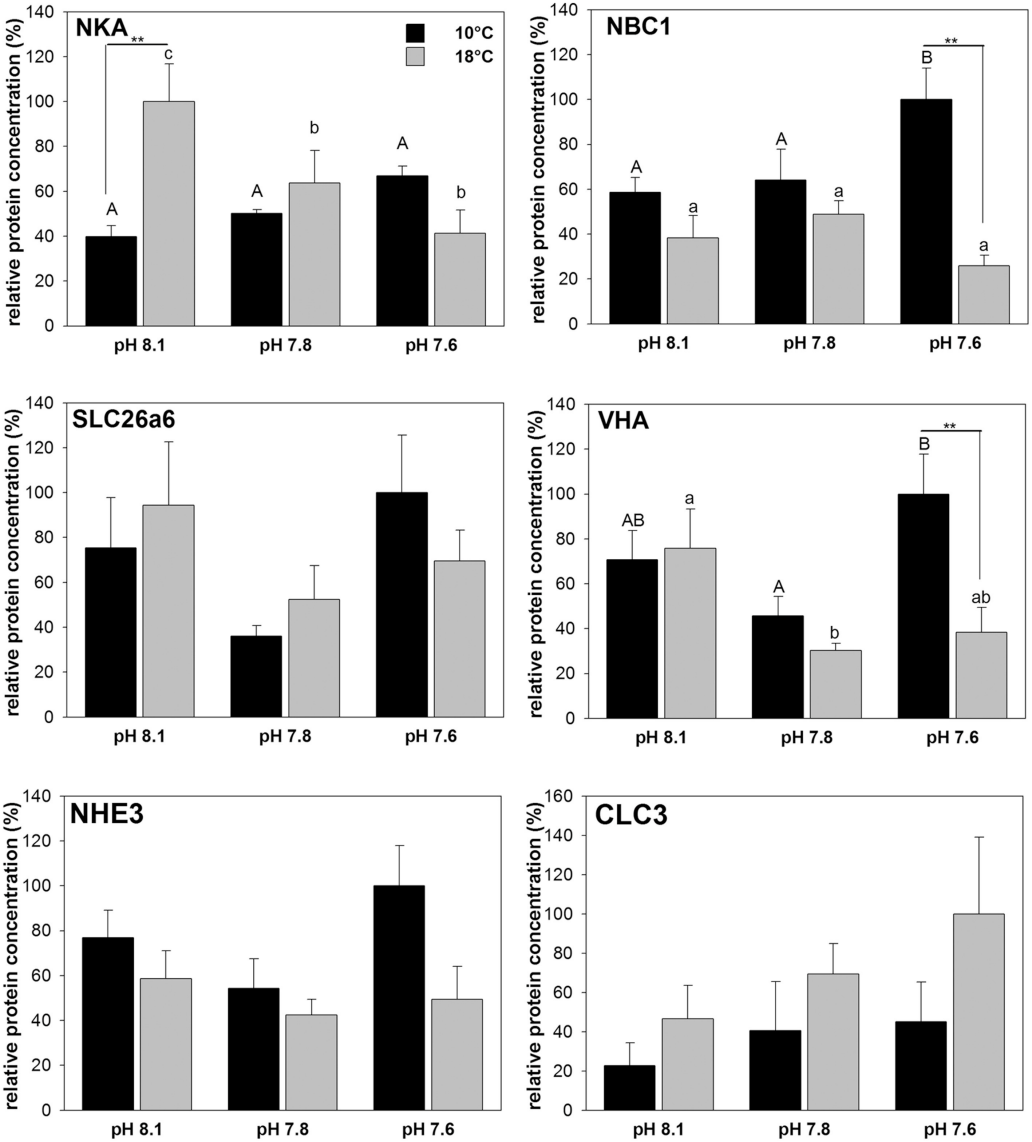
**Relative protein concentrations of six different acid-base relevant transporters in homogenates of anterior intestine tissues from cod acclimated to three different seawater pH and two temperature levels for 4 weeks**. Protein concentrations were determined via western blot analysis and normalized to ß-actin. Different letters denote significant differences between pH treatments whereas asterisks indicate differences between temperature treatments (^**^p < 0.01). Correlation analyses and comparisons between linear regressions at the two acclimation temperatures are presented in Table 4. Values are given as mean ± SE (*n* = 5–6).

Among the tested transporters including NKA, NBC1, SLC26a6, VHA, NHE3, and CLC3 significant differences between temperature and *p*CO_2_ treatments were found for NKA, NBC1, and VHA using two-way ANOVA (Figure 4; Table 3). Hypercapnia had an opposing effect on NKA protein concentration depending on the acclimation temperature. At 10°C NKA protein concentrations were significantly increased in the high *p*CO_2_ (pH 7.6) treatment compared to the low *p*CO_2_ (pH 8.1) and intermediate *p*CO_2_ (pH 7.8) groups, with about 1.5-fold higher expression in high *p*CO_2_ treated animals compared to animals kept at low *p*CO_2_ conditions. NKA protein concentrations decreased with decreasing pH resulting in a 2.5-fold reduction in the high *p*CO_2_ treatment compared to the low *p*CO_2_ group at 18°C. This observation is also reflected in a strong correlation of NKA protein levels at different *p*CO_2_ levels. Correlation analyses (ANCOVA) also demonstrate a significant difference between the linear regressions at different acclimation temperatures (Table 4). A similar increase was observed for NBC1 at 10°C, where protein concentration significantly increased (1.7-fold) in response to elevated seawater *p*CO_2_, (high *p*CO_2_ treatment) whereas no significant differences were observed in the warm acclimated fish. This resulted in significantly (*p* < 0.01) decreased NBC1 protein levels in the 18°C treatment compared to animals kept at 10°C, at high *p*CO_2_ levels. Furthermore the significant correlation between NBC protein levels and *p*CO_2_ at 10°C disappeared in the warm-acclimated animals (Table 4). The intestinal VHA protein concentration increased about 2.4-fold at 10°C, at high compared to intermediate pCO_2_. However, acclimation at 18°C resulted in an opposing effect with decreasing VHA protein concentrations under increasing pCO_2_ conditions (see Table 4 for regression analyses and ANCOVA results). Protein concentrations of SLC26a6, NHE3 and CLC3 were not significantly affected by temperature or by different pCO_2_ levels.

### Gene expression pattern of intestinal acid-base transporters

**Figure 5:**
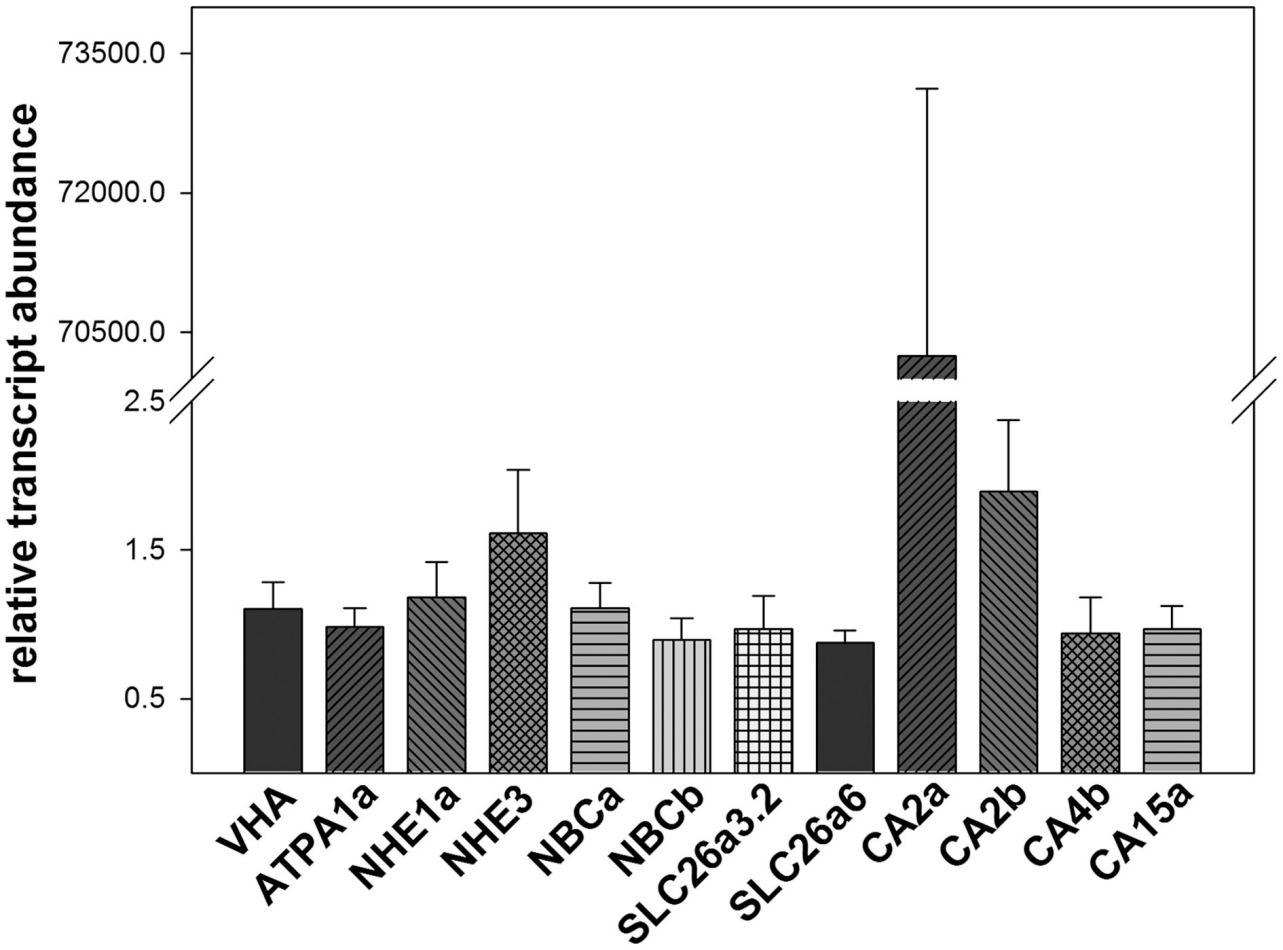
**Transcript abundance of 12 acid-base relevant transporters in the anterior intestine of Atlantic cod ***Gadus morhua*** acclimated to 10°C and 0.05 kPa ***p***CO_**2**_**. Expression levels were normalized to the geometric mean of the housekeeping genes UCE2a and RPL4. Values are given as mean ± SE (*n* = 5–8).

Transcript abundance of 12 acid-base relevant transporters indicated relatively homogenous expression levels of VHA, NKA (ATP1A1), NHE1a, NBCa, NBCb, SLC26a3.2, SLC 26a6 and the carbonic anhydrase isoforms, CA4b and CA15a, in the anterior intestine of Atlantic cod. For NHE3 and the carbonic anhydrase isoform CA2b, 0.5 and 2-fold higher transcript abundances were determined, whereas the carbonic anhydrase isoform CA2a was highly dominating (up to 7000-fold abundance; Figure 5). No detectable transcript abundances could be determined for other acid-base relevant transporters tested including CA4a, CA4c, CA15b, CA15c, VHAb, NHE1b, NHE2, Rhag, Rhcg, AE1a, AE1b, SLC26a3.1, SLC26a5, SLC26a6b, and SLC26a6c (Table S1).

**Figure 6:**
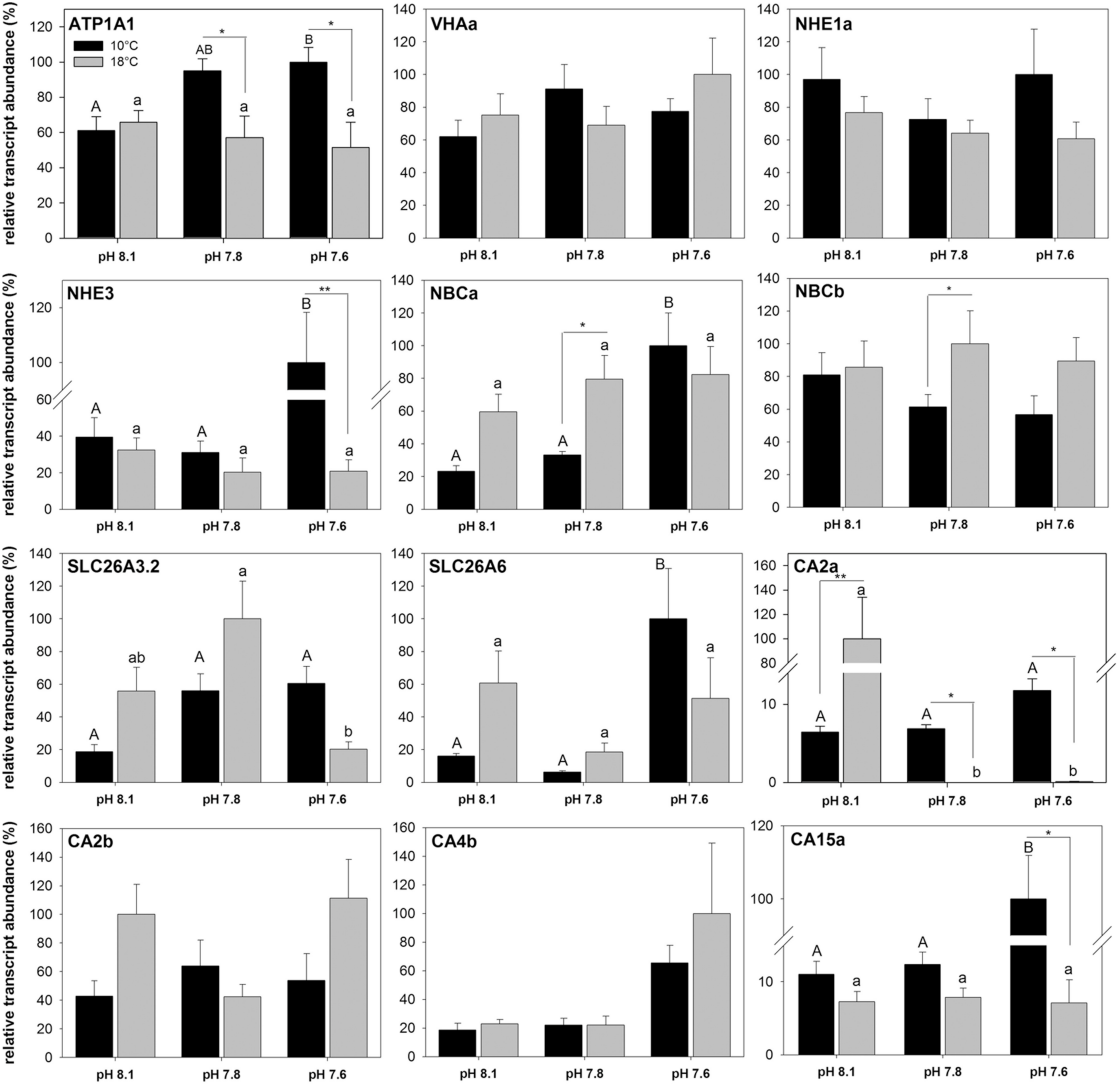
**Relative transcript concentrations of 12 acid-base relevant transporters in the anterior intestine of ***Gadus morhua*** acclimated to three different seawater pH and two temperature levels for 4 weeks**. Expression levels were normalized to the geometric mean of the housekeeping genes UCE2a and RPL4. Different letters denote significant differences between pH treatments whereas asterisks indicate differences between temperature treatments (^*^p < 0.05 and ^**^p < 0.01). Values are given as mean ± SE (*n* = 5–6).

Elevated seawater *p*CO_2_ and increased temperatures affected mRNA expression levels of most intestinal acid-base transporters, including NKA (ATP1A1), NHE3, NBCa, SLC26A3.2, SLC26a6, CA2a, and CA15a (Figure 6). No significant effects of temperature or *p*CO_2_ (pH) were detected for VHAa, NHE1a, NBCb, CA2b, and CA4b. At 10°C, transcript abundance of NKA increased at high *p*CO_2_ conditions compared to animals kept at low *p*CO_2_ (pH 8.1) conditions. No change in transcript levels was observed in response to elevated *p*CO_2_ at 18°C leading to significantly lower transcript levels under elevated seawater *p*CO_2_ conditions in animals acclimated at 18°C. A similar pattern was observed for NHE3, where transcript levels increased about 2.5-fold in response to high *p*CO_2_ at 10°C. No significant CO_2_ effect on NHE3 transcript levels was observed at 18°C, which led to about 4-fold decreased NHE3 mRNA expression levels at 18°C compared to the high *p*CO_2_ treatment at 10°C. NBCa expression responded in a similar fashion as NKA and NHE3 with a pronounced *p*CO_2_ effect on expression levels at 10°C, whereas no *p*CO_2_-dependent change was observed in warm acclimated fish. Transcript levels of the two SLC26 isoforms A3.2 and A6 increased in response to elevated *p*CO_2_ at 10°C, with a significant increase of SLC26A6 expression at high *p*CO_2_. At 18°C, no change was observed for SLC26A6, whereas SLC 26A3.2 transcript levels significantly decreased at high *p*CO_2_ compared to expression levels in the intermediate *p*CO_2_ treatment. A *p*CO_2_ effect on the expression at 10°C was also evident for CA2a and CA15a, where transcript levels significantly increased in response to seawater hypercapnia of 0.2 kPa *p*CO_2_. At 18°C, the carbonic anhydrases showed unchanged CA15a expression but a drastic decrease in expression of the dominating CA2a in response to elevated seawater *p*CO_2_.

Correlation analyses using mRNA levels and protein concentrations of different ion regulatory proteins were used to detect co-regulations on mRNA and protein level during acclimation to different *p*CO_2_ and temperature conditions. Strong correlations (*p* < 0.0001) of mRNA and protein expression were found for NKA and NBCa at 10°C as well as for SLC26A6 and NHE3 at 18°C. Weak correlations were found for VHA at both, 10 and 18°C as well as for NHE3 at 10°C.

## Discussion

### Effects of seawater *p*CO_2_ on intestinal ion transporters

**Figure 7:**
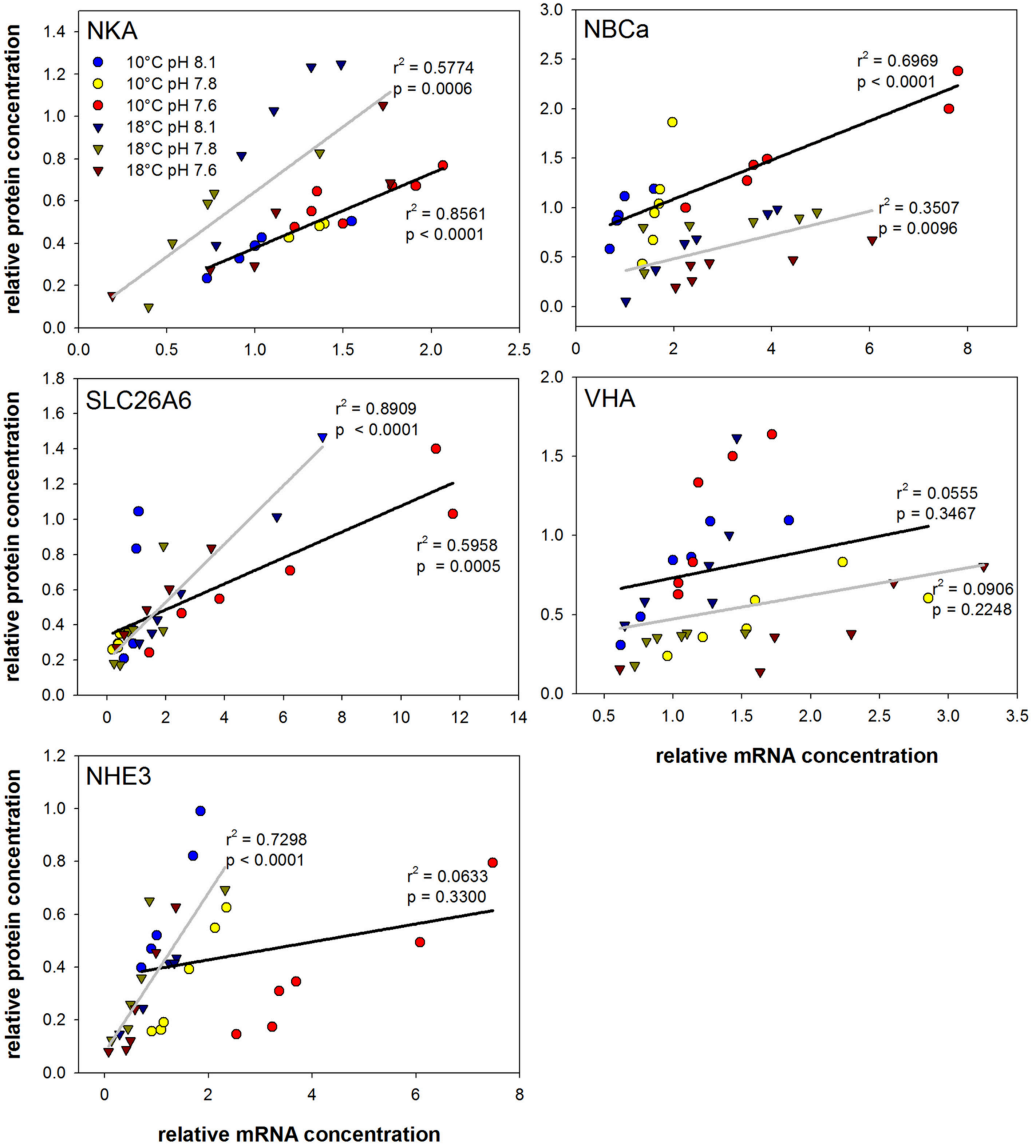
**Correlation plots of mRNA and protein concentrations from intestinal acid-base transporters**. Circles represent values from 10°C acclimated animals whereas triangles indicate values of fish acclimated at 18°C. Different colors indicate the different pH treatments. Linear regressions are presented as black (10°C) and gray (18°C) lines including the respective *r*^2^ and *p*-values (See **Table 5** for slope and intercept comparison statistics).

The current model for intestinal ion regulation and water homeostasis in marine teleosts denotes that luminal bicarbonate secretion involves a set of transporters, including intra- and extracellular carbonic anhydrases, apical SLC26A6, VHA and basolateral NBC1 and NKA (Grosell, 2011). In addition to these confirmed transporters the present study demonstrated the presence of apical NHE3 in the anterior intestine of Atlantic cod. In response to transfer from freshwater to 65% seawater the transcript abundance of NHE3 was significantly up-regulated in intestinal tissues of rainbow trout (*O. mykiss*) (Grosell et al., 2007). Moreover, addition of amiloride to the luminal side increased net ${\text{HCO}}_{3}^{-}$ secretion, suggesting that NHEs located in the apical membranes of the fish intestine may not only contribute to apical Na^+^ absorption (Grosell, 2011) but also to apical H^+^ secretion, titrating some of the secreted bicarbonate (Wilson et al., 1996). Accordingly, the present work underlines a significant contribution of NHE3 to intestinal ion and pH homeostasis by revealing significantly increased mRNA levels in response to high *p*CO_2_. Interestingly, transcript levels of NKA, NBC1, SLC26A6, CA2, and CA15 were also significantly increased in animals from the high *p*CO_2_ treatment at optimum temperature (10°C), suggesting that these transporters operate in concert with each other during environmental hypercapnia. Strong correlation of mRNA and protein levels was found for NKA, NBCa/NBC1 and SLC26A6 at optimum temperature (Figure 7). Thus, a similar transfer of the transcriptomic response to the protein level may be deduced for the other co-regulated proteins (CA2 and CA15), where no information could be obtained due to the lack of available antibodies.

Correlations between mRNA and protein levels were generally weaker at high temperature (Figure 7). This may be indicative for alterations in the translational regulation of intestinal ion transporters (e.g., mismatches between synthesis processes and half-life of the protein) upon exposure to 18°C. Furthermore, correlation analyses between mRNA and protein concentrations of transporters at different hypercapnia levels suggest a strong linear relationship that could depend on the acclimation temperature. NHE3 expression and protein levels are strongly affected by the *p*CO_2_ treatment leading to a weak correlation at 10°C. However, at 18°C NHE3 mRNA and protein levels show a good correlation, reflecting a weak *p*CO_2_ effect on this transporter at this acclimation temperature. The coupling of apical NHE3 activity to ${\text{HCO}}_{3}^{-}$ transport *via* Cl^−^/${\text{HCO}}_{3}^{-}$ exchange has been previously suggested as a potential mechanism to reabsorb Na^+^ and Cl^−^ from the luminal space in the marine teleost intestine (Grosell, 2011). This mechanism is also found in the mammalian intestinal tract, where the coupling of apical membrane Na^+^/H^+^ and Cl^−^/${\text{HCO}}_{3}^{-}$ exchangers has been demonstrated to represent an important mechanism for ion homeostasis and water balance by allowing electroneutral NaCl absorption (Kiela et al., 2006).

The expression patterns observed here are supported by a previous study, demonstrating increased intestinal expression levels of NHE3, NBCa, AE1a, and ATP1A1b (NKA) in seawater acclimated medaka (*Oryzias latipes*) in response to acute exposure (48 h) to a seawater *p*CO_2_ of 0.7 kPa (Tseng et al., 2013). This indicates that also in this euryhaline species, short-term environmental hypercapnia evokes similar responses of the intestinal acid-base regulatory machinery. In addition to the medaka study the present work could demonstrate that these effects were still evident upon long-term (4 weeks) hypercapnic exposure. In contrast to medaka, no expression of AE1 could be detected in intestinal tissues of *G. morhua*, suggesting that this transporter may be differentially expressed in marine and euryhaline teleost species.

**Figure 8:**
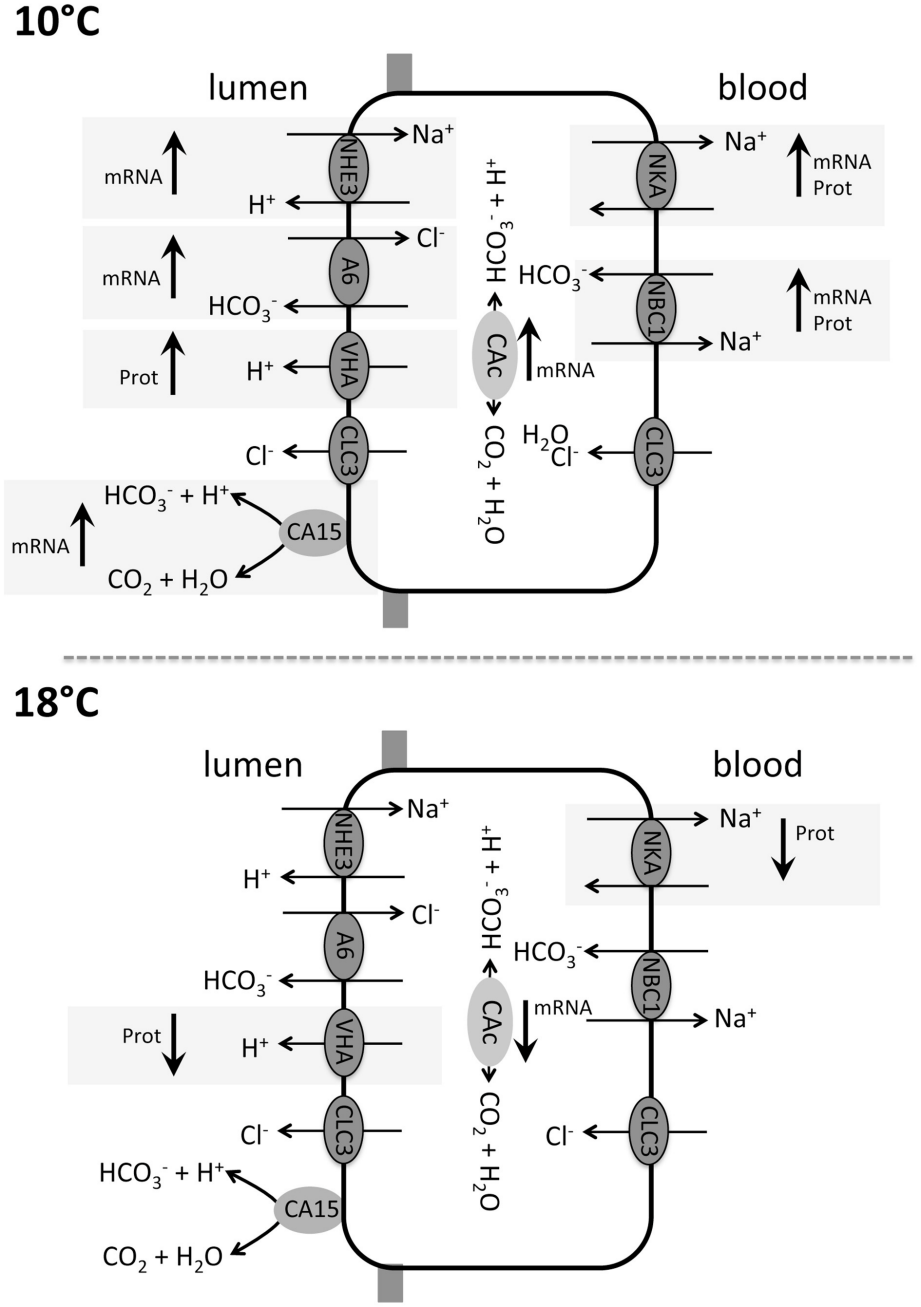
**Intestinal model of acid-base relevant transporters in Atlantic cod ***Gadus morhua***, including differential response patterns toward elevated seawater ***p***CO_**2**_ (decreased pH) in animals acclimated to 10°C and 18°C**. Affected transporters are indicated by a gray background and an arrow indicating up- or down regulation on the mRNA or protein (Prot) level. In animals acclimated to 10°C most transporters revealed an up regulation pattern either on the expression level, protein level or both.

Seawater hypercapnia has been demonstrated to affect intestinal ${\text{HCO}}_{3}^{-}$ secretion rates in several marine teleost species. For example the marine teleost *Porichthys notatus* increased its intestinal ${\text{HCO}}_{3}^{-}$ secretion rates in response to a seawater *p*CO_2_ of 5 kPa (Perry et al., 2010). It was hypothesized that increased blood bicarbonate levels during hypercapnia, necessary for blood pH stabilization, lead to an enhanced loss of ${\text{HCO}}_{3}^{-}$ into the intestinal lumen, whereas protons generated from the intracellular hydration of CO_2_ are exported across basolateral, and probably also, apical membranes (Grosell, 2006, 2011; Perry et al., 2010). Similar findings were obtained for the toadfish (*O. beta*) under prospected future seawater *p*CO_2_ (Heuer et al., 2012). Furthermore, increases in serosal ${\text{HCO}}_{3}^{-}$ levels stimulated NBC1 located in basolateral membranes of enterocytes in toadfish intestines, leading to increased cytosolic ${\text{HCO}}_{3}^{-}$ concentrations (Taylor et al., 2010). Here it has been suggested that increased blood *p*CO_2_ levels induced by environmental hypercapnia reduce the concentration gradient between blood and cytosolic CO_2_, leading to decreased CO_2_ diffusion across intestinal enterocyte membranes and increased cytosolic *p*CO_2_. Such an increased cytosolic [${\text{HCO}}_{3}^{-}$] and *p*CO_2_ can affect the intestinal ion regulatory machinery in different ways: (i) increased cytosolic ${\text{HCO}}_{3}^{-}$ stimulates apical anion exchange and thereby, enhances apical Cl^−^ absorption (Heuer et al., 2012); and (ii) increased cytosolic ${\text{HCO}}_{3}^{-}$ and probably also CO_2_ levels stimulate apical NKCC, mediated through adenylyl cyclase leading to an increased NaCl absorption as demonstrated for the toadfish intestine (Tresguerres et al., 2010). Besides the potential modulation of NKCC, cytosolic adenylyl cyclase stimulated by intracellular ${\text{HCO}}_{3}^{-}$ formation through CAc has been demonstrated to modulate translocation of VHA from vesicles to the plasma membrane in marine fish (Tresguerres et al., 2011). Accordingly, translocation of VHA into apical membranes would increase (together with NHE3) the titration of luminal ${\text{HCO}}_{3}^{-}$ leading to enhanced excretion of ${\text{HCO}}_{3}^{-}$ by the intestine (Figure 8). Here it should be noted that despite stimulated intestinal ${\text{HCO}}_{3}^{-}$ excretion under environmental hypercapnia leading to an increased net export of CO_2_, the major site for CO_2_ excretion driven by the outward directed CO_2_ gradient still occurs via gill epithelia. Furthermore, an up regulation of NKA in branchial epithelia during environmental hypercapnia and 10°C in the same animals as used in the present work suggests that gill epithelia are capable actively countering both, the extracellular acidosis and the increased intestinal loss of ${\text{HCO}}_{3}^{-}$ (Michael et al., 2016b). Besides acid-base disturbances, elevated *p*CO_2_ may thus affect the osmotic balance of marine teleosts by modulating a new steady state between intestinal and gill ion-transport processes. In this context it was proposed that a stimulation of the osmoregulatory machinery during environmental hypercapnia may also affect the drinking rate of marine fish (Ando and Nagashima, 1996). However, exposure to environmental hypercapnia (1900 μatm; 0.2 kPa CO_2_) did not affect the drinking rate in toadfish (Heuer et al., 2012). Moreover, blood plasma osmolarity was unchanged by ambient *p*CO_2_ in 10° acclimated animals, but was increased at 18°C and moderately elevated *p*CO_2_ levels (1200 μatm *p*CO_2_) in the same animals as used for the present work (Kreiss et al., 2015). Furthermore, plasma Cl^−^ levels in these animals significantly decreased with increasing seawater *p*CO_2_ which is probably related to increased anion exchange (${\text{HCO}}_{3}^{-}$ and Cl^−^) activity in branchial epithelia to compensate blood pH (Kreiss et al., 2015). Accordingly, despite a stimulation of intestinal anion exchange and ${\text{HCO}}_{3}^{-}$ secretion by increased *p*CO_2_ branchial epithelia seem to represent the major site for controlling extracellular pH homeostasis in marine teleosts leading to an increased net secretion of Cl^−^ at optimum temperature.

### Regulation of intestinal Na^+^/K^+^-ATPase and V-Type H^+^-ATPase

In the intestinal epithelium of marine teleosts, ion and water balance is controlled by secondary active transporters which are fueled by the electro-chemical gradient of the basolateral NKA (Grosell and Genz, 2006). The present work demonstrates that environmental hypercapnia significantly increases NKA mRNA and protein concentrations in a strongly correlated way, while no change has been detected on the activity level. These seemingly inconsistent results in response to acid-base disturbances may be due to post-translational modifications of the protein. In this respect, reversible phosphorylation of the α -subunit by several protein kinases (PKA, PKC, PKG, tyrosine kinase) was reported to modulate NKA activity in vertebrates and invertebrates (Bertorello and Katz, 1995; Chibalin et al., 1999; Ramnanan and Storey, 2006). Furthermore, FXYD proteins were demonstrated to specifically modulate Na^+^ affinity of NKA in vertebrates (Geering, 2005). This rich diversity of post-translational modifications may explain why the activity of the NKA remains unchanged in response to elevated seawater hypercapnia despite increased mRNA expression levels and protein concentrations. Thus, the presence of higher NKA protein concentrations may potentially increase the ion regulatory capacity of intestinal epithelia allowing for a quick activation of NKA *via* post-translational pathways during acute demands. Interestingly, while CO_2_ had no effects on branchial (Michael et al., 2016b) and intestinal NKA activities temperature evokes opposing effects in these two tissues. While 18°C acclimated animals have uncompensated maximum activities (approximately 40% higher activities at habitat temperature) in gill tissues compared to those kept at 10°C (Michael et al., 2016b) intestinal NKA activities in the same animals are at least partly compensated in warm acclimated animals (40% reduction at a common assay temperature). This suggests that gill and intestinal epithelia use different compensation mechanisms during acclimation to warm temperatures compared to the situation at optimum temperatures (Michael et al., 2016b). Furthermore, the downregulation of NKA activities under elevated temperatures may be explained by the onset of a stress response leading to a release of endogenous ouabain-like substances (Kajimura et al., 2004, 2005). Finally, it should be noted that nutrient absorption after feeding may also induce intestinal transport activity, and thus future studies need to include feeding effects on intestinal transport functions under environmental hypercapnia.

The VHA in intestinal epithelia is predominantly located in apical membranes, where it is thought to energize the formation of intracellular ${\text{HCO}}_{3}^{-}$ by removing protons from the cytosol to the luminal space (Grosell et al., 2009). Our results indicate that similar to NKA, VHA protein concentrations are also increased in response to environmental hypercapnia. However, besides post-translational modifications, our histological analyses demonstrate, that the VHA is also stored in sub-apical vesicles, in order to provide increased capacities in apical membranes, when needed. The post-translational recycling of VHA by trafficking this protein from vesicles to the outer plasma membrane has been extensively studied in mammalian epididymis clear cells, where apical VHA acidifies the lumen for sperm maturation and for their storage in a quiescent state (Pastor-Soler et al., 2007; Shum et al., 2010). Recycling of VHA from vesicles to apical membranes is achieved by the cytoskeleton and controlled by serine/threonine-specific protein kinase cascade mechanisms (Shum et al., 2010). Our results suggest that in the teleost intestine post-translational modification or recycling of VHA from vesicles to plasma membranes may also represent an important mechanism to control intestinal ion regulatory capacities.

### Interaction of elevated temperature and acidification on intestinal acid-base regulation

This work demonstrated that the intestinal ion/acid-base regulatory machinery responds to moderately increased seawater *p*CO_2_ levels by supporting increased ${\text{HCO}}_{3}^{-}$ secretion rates, as observed for other marine teleost species (Wilson et al., 2009; Heuer et al., 2012). In addition, our results also indicate that the intestinal acid-base transport machinery respond very differently to environmental hypercapnia when Atlantic cod were acclimated close to their upper habitat temperature (18°C). Generally, ectothermic animals have a limited thermal window, in which the optimal range is reflected by best physiological performance of an organism. The edges of this window are characterized by a decrease in performance indicated by decreased oxygen availability and a progressive shift to anaerobic metabolism (Pörtner, 2001). It has been hypothesized, that elevated temperatures close to an organisms thermal maximum in combination with hypercapnia, may narrow an organism's thermal window (Pörtner, 2002, 2012; Metzger et al., 2007; Walther et al., 2009). This interplay of temperature and pH stress could be linked to energetic limitations deriving from limited energy acquisition (lowered oxygen transport) and an increased ATP demand to fuel compensatory processes (e.g., intra and extracellular acid-base regulation). In isolated perfused gills of Antarctic fish (*Gobionotothen gibberifrons* and *Notothenia coriiceps*) higher fractions of energy were spent on acid-base regulatory processes in response to acute high CO_2_ treatment (10,000 μatm) (Deigweiher et al., 2010). Similar, higher branchial NKA capacities became visible after long-term acclimation of several fish species, including eelpout and Atlantic cod, indicating higher energetic expenses for ion exchanging processes (Deigweiher et al., 2008; Melzner et al., 2009). In isolated gill tissues from the same animals as used in the present study a higher fraction of energy was allocated to EIPA sensitive Na^+^/H^+^ exchange as well as DIDS sensitive ${\text{HCO}}_{3}^{-}$ transport processes at high *p*CO_2_ and 18°C. However, overall energy demand of perfused gill was unaffected by environmental hypercapnia at this temperature (18°C) leading to an unchanged energy budget of the whole animal (Kreiss et al., 2015). This new energetic equilibrium under increased acid–base regulatory expenses could also partly explain a downregulation pattern of intestinal ion transporters as observed in the present study. Unchanged or even decreased ${\text{HCO}}_{3}^{-}$ secretion activity in response to elevated temperature in combination with elevated seawater CO_2_ levels can be regarded as a possible energy saving mechanism. However, altered gas solubility under different environmental acclimation temperatures may be an additional factor for changes in expression levels of carbonic anhydrases. Also here, energy saving mechanisms could explain the dramatic down regulations in CA2a and CA15a under high environmental *p*CO_2_ (low pH) at 18°C. Additionally, the counter-productive base loss in response to elevated serosal ${\text{HCO}}_{3}^{-}$ levels, which prevents an extracellular acidosis, is likely to be associated with increased branchial acid-base regulatory activity (e.g., ${\text{HCO}}_{3}^{-}$ import and H^+^ export as demonstrated in Kreiss et al., 2015). This assumption is supported by the fact that branchial epithelia represent the major site of H^+^ export and ${\text{HCO}}_{3}^{-}$ import countering respiratory acidosis in marine teleosts (Esbaugh et al., 2012).

## Conclusion

In the present study temperature affected several intestinal acid-base transporters. At optimum temperature, a clear CO_2_ effect became visible at the mRNA and protein level corroborating previous findings (Heuer et al., 2012) that demonstrated enhanced intestinal base secretion in response to moderately increased seawater *p*CO_2_ levels, as prospected for the near future (summarized in Figure 8). In contrast, acclimation to the upper habitat temperature of *G. morhua* led to unchanged or even decreased transcript levels of major intestinal ion transporters during exposure to hypercapnic conditions (Figure 8). Such a potential loss in intestinal ion regulatory capacities can also have severe repercussions on the whole organisms ion homeostasis and water balance, as ${\text{HCO}}_{3}^{-}$ transport is believed to be coupled to salt and water resorption (Grosell, 2011).

As a first step, the present work could show that moderately elevated seawater *p*CO_2_ and temperature are clearly interacting abiotic factors modulating the intestinal ion-regulatory machinery of Atlantic cod. The partly opposing actions of both factors may cause more stressful conditions at the edges of the thermal window. It remains open whether these effects are only visible upon short- to medium-term acclimation or will still be evident in trans-life cycle experiments. Thus, multi-generation experiment in combination with predicted global warming simulations would help to differentiate between physiological plasticity and adaptation potential of intestinal ion transport processes in marine teleosts facing changing environmental conditions on evolutionary short time scales.

## Materials and methods

### Experimental animals and treatments

The animal experiments were conducted according to ethical commission Dnr.: 23-2012, approved by Sweden's Ethical Committee on Animal Experiments. Atlantic cod, *G. morhua* was caught with bow nets during February and March 2012 in the Gullmarsfjord, Sweden and incubated at the Sven Lovén Centre for Marine Sciences, Kristineberg as previously described by Michael et al. (2016b) and Kreiss et al. (2015). For each CO_2_ and temperature treatment, a group of 8–10 animals per replicate (two replicate tanks for each treatment) mixed in gender and size (18.1–37 cm standard length, 76.4–487.4 g) were separately incubated to CO_2_ concentrations of 552.97 ± 78.1 μatm, 1469.93 ± 459.93 μatm, and 2227.95 ± 312.33 μatm at water temperatures of 10.2 ± 0.2°C and 18.1 ± 0.2°C, respectively (for details see Kreiss et al., 2015). The fish were fed *ad libitum* three times a week with frozen shrimps and blue mussels prior and during the incubation period but not the last 48 h before sampling. After 4 weeks of incubation, fish were anesthetized with MS-222 (0.2 g·l^−1^), and length and weight were determined. Fish were killed by a cut through their spine as close to the cranium as possible. Animals were dissected and tissue samples from the anterior intestine were cleaned and immediately shock frozen in liquid nitrogen and stored at −80°C for RNA and protein extraction. Samples for immunohistochemical analyses were fixed in Bouin's solution over-night, and stored in 75% ethanol.

### Immunohistochemical localization of intestinal acid-base transporters

For immunohistochemistry, tissues were fixed by direct immersion in Bouin's fixative for 24 h followed by rinses in 70% ethanol. Samples were fully dehydrated in a graded ethanol series and embedded in Paraplast (Paraplast Plus, Sigma, P3683). Sections of 4 μm were cut on a Leitz Wetzlar microtome, collected on poly-L-lysine-coated slides, and stored at 37°C for 48 h. The slides were deparaffinized in Histoclear II® for 10 min and passed through a descending alcohol series (100, 95, 90, 70, and 50% for 5 min each). Slides were washed in phosphate- buffered saline (PBS), pH 7.3. Subsequently, samples were transferred to a PBS solution containing 5% bovine serum albumin (BSA) for 30 min to block non-specific binding. The primary antibodies (Table 1), a mouse monoclonal antibody IgG α5, raised against the avian α subunit of the Na^+^/K^+^-ATPase (Developmental Studies Hybridoma Bank, University of Iowa, USA), a polyclonal antibody raised against NBC1 of Atlantic cod (Michael et al., 2016b), a polyclonal antibody raised against a synthetic peptide corresponding to a COOH terminal region of tilapia NHE3 (generously provided by Dr. Toyoji Kaneko, University of Tokyo), a polyclonal antibody raised against SLC26a6 (pendrin) of zebrafish (*Danio rerio*) (generously provided by Dr. Pung-Pung Hwang, Academia Sinica), a polyclonal antibody raised against the V-type H^+^-ATPase of squid (*Sepioteuthis lessoniana*) (Hu et al., 2013), a polyclonal antibody raised against an intracellular region of rat CLC3 (Alomone labs Ltd, Jerusalem, Israel) were diluted in PBS to 5–10 μg ml^−1^ and placed in small droplets of 100 μl onto the sections, and incubated for 12 h at 4°C in a wet chamber. To remove unbound antibodies, the sections were then washed (3 × 5 min) in PBS and incubated for 1 h with small droplets (100 μl) of secondary antibody, anti-mouse alexafluor 488 or anti- rabbit alexafluor 568 (Invitrogen Oregon, USA). After rinses in PBS (3 × 5 min), sections were examined with a fluorescence microscope (Zeiss imager A1) with an appropriate filter set and a phase-contrast device.

**Table 1:** **Description of antibodies used for immunohistochemical localization of acid-base transporters in the cod intestine**.

**Antibody**	**Abbreviation**	**Color code**	**Description**	**Species**	**Host**
Na^+^/K^+^-ATPase	NKA	Green	Raised against the avian α subunit	Chicken	Mouse
Na^+^/${\text{HCO}}_{3}^{-}$ -cotransporter 1	NBC1	Red	Designed against peptide sequence EKEPFLGDKSFDK, (COOH terminal region)	Cod	Rabbit
Na^+^/H^+^-exchanger 3	NHE3	Turquois	Designed against synthetic peptide sequence TDTKQMNNDQFPPP, (COOH terminal region)	Tilapia	Rabbit
Na^+^-dependent Cl^−^/${\text{HCO}}_{3}^{-}$ -antiporter	SLC26a6	Yellow	Designed against peptide sequence RLKERSQRMNPSQIC	Zebrafish	Rabbit
V-type H^+^-ATPase	VHA	Purple	Designed against synthetic peptides corresponding to the subunit A region (SYSKYTRALDEFYDK)	Squid	Rabbit
Cl^−^ channel 3	CLC3	Blue	Designed against synthetic peptides corresponding to residues 592-661 near the COOH terminal region	Rat	Rabbit

### Na^+^/K^+^-ATPase activity assay

Na^+^/K^+^-ATPase activity was measured in crude extracts in a coupled enzyme assay with pyruvate kinase (PK) and lactate dehydrogenase (LDH). Crude extracts were obtained by quickly homogenizing the tissue samples in a conical tissue grinder in 10 volumes of ice-cold buffer containing 50 mM imidazole, pH 7.8, 250 mM sucrose, 1 mM EDTA, 5 mM β-mercaptoethanol, 0.1% (w/v) deoxycholate, proteinase inhibitor cocktail from Sigma-Aldrich (Taufkirchen, Germany; catalog no. P8340). Extraction was performed on ice to avoid protein degradation. Cell debris was removed by centrifugation for 10 min at 1000 *g*, 4°C. The supernatant was used as a crude extract. The reaction was started by adding 1.5 μl of the sample homogenate to the reaction buffer containing 100 mM imidazole, pH 7.8, 80 mM NaCl, 20 mM KCl, 5 mM MgCl_2_, 5 mM ATP, 0.24 mM Na-NADH_2_, 2 mM phosphoenolpyruvate, and about 12 U/ml PK and 17 U/ml LDH in a PK/LDH enzyme mix (Sigma-Aldrich). The oxidation of NADH coupled to the hydrolysis of ATP was followed photometrically at 25°C in a temperature controlled plate reader (VictorX, Perkin Elmer) over a period of 15 min with the decrease of extinction being measured at λ = 339 nm. The fraction of Na^+^/K^+^-ATPase activity in total ATPase (TA) activity was determined by the addition of ouabain to a final concentration of 5 mM to the assay. Each sample was measured in three replicates with and without the addition of ouabain. Enzyme activity was calculated by using the extinction coefficient for NADH of ε = 6.31 mM^−1^·cm^−1^ and given as micromoles of ATP consumed per mg protein per hour.

### Immunoblotting

10 μL of crude tissue extract were fractionated by SDS-PAGE on 10% polyacrylamide gels, according to Lämmli (1970), and transferred to PVDF membranes (Millipore), using a tank blotting system (Bio-Rad). Blots were pre-incubated for 1 h at room temperature in TBS-Tween buffer (TBS-T, 50 mM Tris -HCl, pH 7.4, 0.9% (wt/vol) NaCl, 0.1% (vol/vol) Tween20) containing 5% (wt/vol) nonfat skimmed milk powder to quench unspecific protein binding. Blots were incubated with the primary antibody (see previous section) diluted 1:250–500 at 4°C overnight. After washing with TBS-T, blots were incubated for 2 h with horseradish conjugated goat anti-rabbit/mouse IgG antibody (diluted 1:2000, at room temperature; Amersham Pharmacia Biotech). Protein signals were visualized by using the enhanced chemiluminescence system (ECL, Amersham Pharmacia Biotech) and recorded using Biospectrum 600 imaging system (UVP, Upland, CA, USA). Protein band intensities were analyzed using the free image analysis software ImageJ (Schneider et al., 2012).

### Preparation of mRNA

Anterior intestine tissue (without pyloric caecae) was homogenized in Trizol reagent (Invitrogen, Carlsbad, CA, USA) using the Tissue Lyser (Quiagen). Total RNA was purified using the RNeasy Mini Kit (Quiagen) following the manufacturer's protocol and DNA contamination was removed with DNase I (Promega, Madison, WI, USA). The amount of mRNA was determined by spectrophotometry (ND-2000, NanoDrop Technol, Wilmington, USA), and the RNA integrity was checked by electrophoresis in RNA denatured gels (Sambrook et al., 1987). All mRNA pellets were stored at −80°C.

### Real-time quantitative PCR (qRT-PCR)

Using the cod genome (http://www.codgenome.no/), we selected sequence information of 29 acid-base transporters. Primers for all genes were designed (Table 2) using Primer Premier software (vers. 5.0; PREMIER Biosoft International, Palo Alto, CA, USA). The mRNA expression of target genes was measured by qRT-PCR with the Roche LightCycler® 480 System (Roche Applied Science, Mannheim, Germany). PCRs contained 3.2 ng cDNA, 50 nM of each primer, and the LightCycler® 480 SYBR Green I Master Mix (Roche) in a final volume of 10 μL. All PCRs were performed as follows: 1 cycle of 50°C for 2 min and 95°C for 10 min, followed by 40 cycles of 95°C for 15 s and 60°C for 1 min (the standard annealing temperature of all primers). PCR products were subjected to a melting-curve analysis to verify that only a single product was present. Control reactions were conducted with sterile water to determine levels of background and genomic DNA contamination. The standard curve of each gene was confirmed to be in a linear range with ribosomal protein L4 (RPL4) and ubiquitin conjugated enzyme 2a (UCE2a) which were used as reference genes. The expression of ribosomal protein and ubiquitin conjugated enzyme homologs have been demonstrated to be stable among ontogenetic stages and during hypercapnic exposure in teleosts (Tseng et al., 2013) and cephalopods (Hu et al., 2013, 2014).

**Table 2:** **Primers used for qRT-PCR**.

**Gene name**	**Abbreviation**	**Primer sequence**	**Amplicon size (bp)**	**Accession numbers**
V-Type H^+^-ATPase a	VHAa	F 5′-GACAAGCACTTCCCAGAGTT-3′	134	ENSGMOT00000017186
		R 5′-TCCAGGGTGATCTTATCCGT-3′		
V-Type H^+^-ATPase b	VHAb	F 5′-GCCCTGAACAGAGACATCAA-3′	130	ENSGMOT00000002718
		R 5′-GGAGCATCAGCTTGTGTTTG-3′		
Na^+^/K^+^-ATPase ATP1a1	ATP1a1	F 5′-GTCACCATCCTCTGCATTGA-3′	132	ENSGMOT00000005767
		R 5′-CCTCTCGTTGACCAGTTTGT-3′		
Na^+^/H^+^-exchanger 1a	NHE1a	F 5′-CTTCGAGGAGATCCACATCAAC-3′	103	ENSGMOT00000004366
		R 5′-CTCGTCAAACAGGTGGTACAG-3′		
Na^+^/H^+^-exchanger 1b	NHE1b	F 5′-GCGTTCTCTTGATCGTGTTTG-3′	137	ENSGMOT00000013214
		R 5′-GGTTCTCACGTGATCGTAGTT-3′		
Na^+^/H^+^-exchanger 2	NHE2	F 5′-CTTTGCCATCTCCTCCATCAT-3′	145	ENSGMOT00000013830
		R 5′-GAAGATGAGCGTTTCCGAGAT-3′		
Na^+^/H^+^-exchanger 3	NHE3	F 5′-TGCTGGAGAAGAGCAAGATAAA-3′	155	ENSGMOT00000014326
		R 5′-GATGAGCGAGAGATCTGAGTTG-3′		
Anion exchanger 1a	AE1a	F 5′-CTTCTTTGCGTTCTGCAAGTC-3′	145	ENSGMOT00000001730
		R 5′-CTTGGGTACCATCAACAGGAG-3′		
Anion exchanger 1b	AE1b	F 5′-GGTGATGTTGGACTGTAAGGAG-3′	128	ENSGMOT00000001272
		R 5′-GGTTGTGGATCAGAGAGTTGAG-3′		
Na^+^/${\text{HCO}}_{3}^{-}$ exchanger 1	NBC1	F 5′-CATGAAGAAGTTGCCCAGAGA-3′	169	ENSGMOT00000015497
		R 5′-TGGGCCAAGGAGAATGAATAG-3′		
Na^+^/${\text{HCO}}_{3}^{-}$ exchanger 2	NBC2	F 5′-CACATCGACTCCCTGAAGATG-3′	157	ENSGMOT00000008943
		R 5′-TTGGGATGAACTTGAGGATGG-3′		
SLC26a3.1	A3.1	F 5′-GATTCCTTCGCCCATCTTCTT-3′	151	ENSGMOT00000014438
		R 5′-CCATTCCATGTCTCCGTTCTT-3′		
SLC26a3.2	A3.2	F 5′-GGAGGTCAATGACACGTACAA-3′	143	ENSGMOT00000014414
		R 5′-CCCTCTTCATCTCACCAACAA-3′		
SLC26a5	A5	F 5′-GAGATCATCGTGGTCATCGT-3′	141	ENSGMOT00000003639
		R 5′-GATTCTGGGAAACAGGTGGA-3′		
SLC26a6a	A6a	F 5′-CTACCACAAGGTATGGCCTATG-3′	105	ENSGMOT00000019285
		R 5′-GGAGGTCCCAAAGATGAAGTAG-3′		
SLC26a6b	A6b	F 5′-CCGTGTAGACATGTGTGTGT-3′	126	ENSGMOT00000002392
		R 5′-CTTGGTAGCTGGGTCCTAAAG-3		
SLC26a6c	A6c	F 5′-GACCCAGCTGCCAACTTATT-3′	131	ENSGMOT00000010364
		R 5′-AGTACACCGTGGAAGAGGAA-3′		
Carbonic anhydrase 2a	CA2a	F 5′-ACGGACATTCCTTCCAAGTG-3′	103	ENSGMOT00000017293
		R 5′-TGGAAGTGGAACTGCTTGAG-3′		
Carbonic anhydrase 2b	CA2b	F 5′-GAACGTTCTCTGAAGCCACTTA-3′	124	ENSGMOT00000017257
		R 5′-CGGCAGTCAACGTAGAAGTATT-3′		
Carbonic anhydrase 4a	CA4a	F 5′-GCACCAAATAGCAACACCAG-3′	147	ENSGMOT00000015251
		R 5′-GTGTTCAAACAGGGTCCAGA-3′		
Carbonic anhydrase 4b	CA4b	F 5′-CAGTCGCAGGTGTCATGTAA-3′	145	ENSGMOT00000017884
		R 5′-TGTAACGGAACCAGCCTTTC-3′		
Carbonic anhydrase 4c	CA4c	F 5′-AGTAGCTTGGACGGTGTTTC-3′	140	ENSGMOT00000006568
		R 5′-CCAGTAGACAACTCTGCCATC-3′		
Carbonic anhydrase 15a	CA15a	F 5′-GTGAACGTTAAGGCGATCTACA-3′	131	ENSGMOT00000007252
		R 5′-TAGTACTTGGTACGGTCCACTC-3′		
Carbonic anhydrase 15b	CA15b	F 5′-GCTCCGAACACATGCTGAA-3′	120	ENSGMOT00000002626
		R 5′-TGGTCAGGGAACCCATGTA-3′		
**REFERENCE GENES**				
Ribosomal protein L4	RPL4	F 5′-CTCCACCATCAAGATCAACTAC-3′	160	ENSGMOT00000009188
		R 5′-CTTCAGGTTCTTCAGAGGATTC-3′		
Ubiquitin conjugated enzyme 2a	UCE2a	F 5′-CATATTTGGACCAGAGGGAAC-3′	152	ENSGMOG00000001702
		R 5′-CTAAACATATGCTGCCATCGG-3′		

*F, forward primer; R, reverse primer*.

**Table 3:** **Analysis of variance (ANOVA) results of protein and mRNA concentrations of intestinal ion transporters under different ***p***CO_**2**_ conditions (pH 8.1; 550 μatm, pH 7.8; 1200 μatm and pH 7.6; 2200 μatm at the two temperatures of 10 and 18°C**.

**Protein**	**Two-way ANOVA**				**Comparisons for factor**			
	**Treatment**	***df***	***F***	***P***	**Factor**		***t***	***P***
NKA	*p*CO_2_	2, 25	1.202	0.317	18°C	8.1 vs. 7.6	3.770	0.003^*^
	T	1, 25	3.248	0.084		8.1 vs. 7.8	2.432	0.045^*^
	*p*CO_2_ × T	2, 25	7.909	0.002^*^		7.6 vs. 7.8	1.506	0.145
NBC	*p*CO_2_	2, 29	1, 178	0.322	10°C	7.6 vs. 8.1	3.045	0.015^*^
	T	1, 29	23, 16	<0.001^*^		7.6 vs. 7.8	2.768	0.019^*^
	*p*CO_2_ × T	2, 29	6, 270	0.005^*^		7.8 vs. 8.1	0.405	0.688
VHA	*p*CO_2_	2, 30	5, 383	0.010^*^	10°C	7.6 vs. 7.8	3.260	0.008^*^
	T	1, 30	6, 231	0.018^*^		7.6 vs. 8.1	1.758	0.170
	*p*CO_2_ × T	2, 30	4, 215	0.024^*^		8.1 vs. 7.8	1.501	0.144
					18°C	8.1 vs. 7.8	2.741	0.030^*^
						8.1 vs. 7.6	2.252	0.063
						7.6 vs. 7.8	0.489	0.628
SLC26A6	*p*CO_2_	2, 28	3, 176	0.057				
	T	1, 28	0.013	0.912				
	*p*CO_2_ × T	2, 28	1, 072	0.356				
NHE3	*p*CO_2_	2, 28	1, 565	0.227				
	T	1, 28	2, 833	0.103				
	*p*CO_2_ × T	2, 28	1, 581	0.224				
CLC3	*p*CO_2_	2, 28	1, 393	0.265				
	T	1, 28	3, 856	0.060				
	*p*CO_2_ × T	2, 28	0.285	0.754				
**mRNA**								
CA2a	*p*CO_2_	2, 24	13.61	0.006	18°C	8.1 vs. 7.8	9.089	<0.001^*^
	T	1, 24	9.173	<0.001^*^		8.1 vs. 7.6	9.079	<0.001^*^
	*p*CO2 × T	2, 24	15.26	<0.001^*^		7.6 vs. 7.8	0.011	1
CA2b	*p*CO_2_	2, 36	1.211	0.053				
	T	1, 36	4.019	0.310				
	*p*CO2 × T	2, 36	2.798	0.074				
CA4b	*p*CO_2_	2, 32	8.367	0.361				
	T	1, 32	0.859	0.001^*^				
	*p*CO_2_ × T	2, 32	0.583	0.564				
CA15	*p*CO_2_	2, 34	35.72	<0.001^*^	10°C	7.6 vs. 8.1	14.66	<0.001^*^
	T	1, 34	44.53	<0.001^*^		7.6 vs. 7.8	14.45	<0.001^*^
	*p*CO_2_ × T	2, 34	36.45	<0.001^*^		7.8 vs. 8.1	0.202	0.989
VHAa	*p*CO_2_	2, 36	1.011	0.374				
	T	1, 36	0.159	0.693				
	*p*CO_2_ × T	2, 36	1.520	0.232				
ATP1A1	*p*CO_2_	2, 31	0.939	0.402	10°C	7.6 vs. 8.1	3.693	0.036^*^
	T	1, 31	10.27	0.003^*^		7.6 vs. 7.8	0.497	0.934
	*p*CO_2_ × T	2, 31	3.597	0.039^*^		7.8 vs. 8.1	3.335	0.063
NHE1a	*p*CO_2_	2, 37	0.734	0.487				
	T	1, 37	3.110	0.086				
	*p*CO_2_ × T	2, 37	0.500	0.611				
NHE3	*p*CO_2_	2, 35	6.267	0.005^*^	10°C	7.6 vs. 7.8	7.099	<0.001^*^
	T	1, 35	15.15	<0.001^*^		7.6 vs. 8.1	5.792	<0.001^*^
	*p*CO_2_ × T	2, 35	8.039	0.001^*^		8.1 vs. 7.8	0.836	0.826
NBCa	*p*CO_2_	2, 35	7.144	0.003^*^	10°C	7.6 vs. 8.1	5.466	0.001
	T	1, 35	4.030	0.052		7.6 vs. 7.8	4.927	0.004
	*p*CO_2_ × T	2, 35	3.356	0.046^*^		7.8 vs. 8.1	0.745	0.859
NBCb	*p*CO_2_	2, 35	0.297	0.745				
	T	1, 35	4.980	0.032^*^				
	*p*CO_2_ × T	2, 35	0.832	0.444				
26A3.2	*p*CO_2_	2, 28	5.317	0.011^*^	18°C	7.8 vs. 7.6	5.715	0.001^*^
	T	1, 28	1.451	0.238		7.8 vs. 8.1	3.325	0.065
	*p*CO_2_ × T	2, 28	5.875	0.007^*^		8.1 vs. 7.6	2.545	0.188
26A6a	*p*CO_2_	2, 31	6.029	0.006^*^	10°C	7.6 vs. 7.8	4.947	0.004^*^
	T	1, 31	0.024	0.878		7.6 vs. 8.1	4.582	0.008^*^
	*p*CO_2_ × T	2, 31	3.405	0.046^*^		8.1 vs. 7.8	0.574	0.914
Rhbg	*p*CO_2_	2, 30	6.417	0.005	18°C	7.6 vs. 8.1	6.080	<0.001^*^
	T	1, 30	6.736	0.014		7.6 vs. 7.8	6.277	<0.001^*^
	*p*CO_2_ × T	2, 30	6.416	0.005		7.8 vs. 8.1	0.011	1

* *Indicates significant (p < 0.05) differences*.

**Table 4:** **Regression analyses for the ***p***CO_**2**_-dependent protein concentrations of intestinal ion transporters in ***Gadus morhua***, and analysis of covariance (ANCOVA) between regressions at the two acclimation temperatures of 10 and 18°C**.

**Parameter**	**Regression**	***R*^2^**	***df***	***F***	***p***
**NKA**					
*p*CO_2_ vs. protein					
10°C	Protein = 0.000158*X + 0.3068	0.641	1, 13	23.21	0.0003^*^
18°C	Protein = 0.0003419*X + 1.05	0.378	1, 14	8.50	0.0113^*^
10°C vs. 18°C _slope_			1, 27	16.95	0.0003^*^
**NBC**					
10°C	Protein = 0.0004158*X + 0.6838	0.2963	1, 15	6.316	0.0239^*^
18°C	Protein = 0.0001224*X + 0.7476	0.0838	1, 16	1.464	0.2439
10°C vs. 18°C _slope_			1, 31	7.959	0.0083^*^
**SLC26A6**					
10°C	Protein = 0.0001245*X + 0.3591	0.0525	1, 15	0.924	0.3766
18°C	Protein = 0.0001222*X + 0.6695	0.0581	1, 15	0.9244	0.3516
10°C vs. 18°C_slope_			1, 30	1.748	0.1961
10°C vs. 18°C _intercept_			1, 31	0.0167	0.8975
**VHA**					
10°C	Protein = 0.0001972*X + 0.5609	0.1103	1, 16	1.983	0.1782
18°C	Protein = 0.0002569*X + 0.8379	0.2355	1, 16	4.928	0.0412^*^
10°C vs. 18°C_slope_			1, 32	6.249	0.0177^*^
**NHE3**					
10°C	Protein = 0.0001566*X + 0.6375	0.1699	1, 15	3.07	0.1002
18°C	Protein = 0.0000414*X + 0.376	0.0185	1, 15	0.2831	0.6025
10°C vs. 18°C_slope_			1, 30	0.9581	0.3355
10°C vs. 18°C _intercept_			1, 31	2.371	0.1337
**CLC3**					
10°C	Protein = 0.0001129*X + 0.1674	0.0442	1, 15	0.6037	0.4180
18°C	Protein = 0.0002753*X + 0.2697	0.1351	1, 15	2.343	0.1467
10°C vs. 18°C_slope_			1, 30	0.5198	0.4765
10°C vs. 18°C _intercept_			1, 31	4.381	0.0446^*^

* *Indicates significant (p < 0.05) differences*.

### Statistical analyses

Values are presented as the mean ± standard error (SE). Statistical analyses were performed on biological samples (*n* = 5–8) acclimated to the respective temperature and *p*CO_2_ conditions in two experimental replicates. Enzyme activities, protein levels and transcript levels were compared using two-way analysis of variance (ANOVA) followed by Tukey's *Post-Hoc* tests (Tukey's *Post-Hoc* with variable *n* = 5–8). Statistical analysis of qRT-PCR results was performed on mRNA quantities normalized to the geometric mean of the housekeeping genes RPL4 and UCE2a (Statistical results for protein and mRNA concentrations are listed in Table 5). Simple linear regression models were used to test the relationship between mRNA/protein concentrations and pH treatment as well as between mRNA and protein concentrations. Analysis of covariance (ANCOVA) was used to test for differences in slope and intercept between mRNA and protein concentration correlations of intestinal ion transporters in *Gadus morhua* acclimated to 10 and 18°C (Table 5). All statistical analyses were performed using Sigma Stat 10.0 (Systat Software). Asterisks indicate significant differences between the two temperature treatments, whereas different letters indicate differences between the three CO_2_ treatments with significance levels of *p* < 0.05 (^*^) and *p* < 0.01(^**^).

**Table 5:** **Results for the regression analyses and analysis of covariance (ANCOVA) between correlations of ***p***CO_**2**_-dependent mRNA and protein concentrations of intestinal ion transporters in ***Gadus morhua*** acclimated to 10 and 18°C**.

**Parameter**	**Regression**	***R*^2^**	***df***	***F***	***p***
**NKA**					
mRNA vs. protein					
10°C	Protein = 0.353*X + 0.0253	0.856	1, 13	77.35	<0.0001^*^
18°C	Protein = 0.615*X + 0.0289	0.577	1, 14	19.13	0.0006^*^
10°C vs. 18°C _slope_			1, 27	2.45	0.1295
10°C vs. 18°C _intercept_			1, 28	19.26	0.0002^*^
**NBC**					
10°C	Protein = 0.198*X + 0.693	0.697	1, 15	34.50	<0.0001^*^
18°C	Protein = 0.120*X + 0.244	0.351	1, 16	8.64	0.0096^*^
10°C vs. 18°C _slope_			1, 31	1.99	0.1684
10°C vs. 18°C _intercept_			1, 32	53.19	<0.0001^*^
**SLC26A6**					
10°C	Protein = 0.074*X + 0.340	0.596	1, 14	20.63	0.0005^*^
18°C	Protein = 0.165*X + 0.200	0.891	1, 15	122.30	<0.0001^*^
10°C vs. 18°C_slope_			1, 29	11.58	0.002^*^
**VHA**					
10°C	Protein = 0.176*X + 0.557	0.056	1, 16	0.94	0.3467
18°C	Protein = 0.151*X + 0.321	0.091	1, 16	1.60	0.2248
10°C vs. 18°C_slope_			1, 32	0.03	0.911
10°C vs. 18°C _intercept_			1, 33	4.65	0.03846^*^
**NHE3**					
10°C	Protein = 0.034*X + 0.360	0.063	1, 15	1.01	0.3300
18°C	Protein = 0.301*X + 0.079	0.730	1, 15	40.51	<0.0001^*^
10°C vs. 18°C_slope_			1, 30	8.98	0.0055^*^

* *Indicates significant (p < 0.05) differences*.

## Author contributions

MH, KM, and CK designed and conducted the experiments of the present work and wrote the manuscript. ML, MS, and SD helped with the experimental design, data analysis and performed statistical analyses. YT conducted gene expression analyses and helped with the immunocytochemical staining. All authors equally contributed to the writing and completion of the manuscript.

### Conflict of interest statement

The authors declare that the research was conducted in the absence of any commercial or financial relationships that could be construed as a potential conflict of interest.

## References

[B1] Ando M. Nagashima C. (1996). Intestinal Na^+^ and Cl^−^ levels control drinking behavior in the seawater-adapted eel *Anguilla japonica*. J. Exp. Biol. 199, 711–716. 9318466 10.1242/jeb.199.3.711

[B2] Bertorello A. M. Katz A. I. (1995). Regulation of Na^+^-K^+^-pump activity: pathways between receptors and effectors. News Physiol Sci. 10, 253–259.

[B3] Chibalin A. V. Ogimoto G. Pedemonte C. H. Pressley T. A. Katz A. I. Féraille E.. (1999). Dopamine-induced endocytosis of Na^+^/K^+^-ATPase is initiated by phosphorylation of Ser-18 in the rat α subunit and is responsible for the decreased activity in epithelial cells. J. Biol. Chem. 274, 1920–1927. 9890946 10.1074/jbc.274.4.1920

[B4] Deigweiher K. Hirse T. Bock C. Lucassen M. Pörtner H. O. (2010). Hypercapnia induced shifts in gill energy budbets of Antarctic notothenoids. J. Comp. Physiol. B. 180, 347–359. 10.1007/s00360-009-0413-x 19834716

[B5] Deigweiher K. Koschnick N. Pörtner H.-O. Lucassen M. (2008). Acclimation of ion regulatory capacities in gills of marine fish under environmental hypercapnia. Am. J. Physiol. 295, R1660–R1670. 10.1152/ajpregu.90403.2008 18799636

[B6] Dixson D. L. Munday P. L. Jones G. P. (2010). Ocean acidification disrupts the innate ability of fish to detect predator olfactory cues. Ecol. Lett. 13, 68–75. 10.1111/j.1461-0248.2009.01400.x 19917053

[B7] Dorey N. Lancon P. Thorndyke M. Dupont S. (2013). Assessing physiological tipping point of sea urchin larvae exposed to a broad range of pH. Glob. Change. Biol. 19, 3355–3367. 10.1111/gcb.12276 23744556

[B8] Esbaugh A. J. Heuer R. M. Grosell M. (2012). Impacts of ocean acidification on respiratory gas exchange and acid-base balance in a marine teleost, *Opsanus beta*. J. Comp. Physiol. B. 182, 921–934. 10.1007/s00360-012-0668-5 22581071

[B9] Geering K. (2005). Function of FXYD proteins, regulators of Na, K-ATPase. J. Bioenerg. Biomembr. 37, 387–392. 10.1007/s10863-005-9476-x 16691470

[B10] Gräns A. Jutfeld F. Sandblom E. Jönsson E. Winklander K. Seth H.. (2014). Aerobic scope fails to explain the detrimental effects on growth resulting from warming and elevated CO_2_ in Atlantic halibut. J. Exp. Biol. 217, 711–717. 10.1242/jeb.096743 24574386

[B11] Grosell M. (2006). Intestinal anion exchange in marine fish osmoregulation. J. Exp. Biol. 209, 2813–2817. 10.1242/jeb.02345 16857865

[B12] Grosell M. (2011). Intestinal anion exchange in marine teleosts is involved in osmoregulation and contributes to the oceanic inorganic carbon cycle. Acta. Physiol. 202, 421–434. 10.1111/j.1748-1716.2010.02241.x 21362153

[B13] Grosell M. Genz J. (2006). Ouabain-sensitive bicarbonate secretion and acid absorption by the marine teleost fish intestine play a role in osmoregulation. Am. J. Physiol. Regul. Integr. Comp. Physiol., 291, R1145–R1156. 10.1152/ajpregu.00818.2005 16709644

[B14] Grosell M. Genz J. Taylor J. R. Perry S. F. Gilmour K. M. (2009). The involvement of H^+^-ATPase and cerbonic anhydrase in intestinal ${\text{HCO}}_{3}^{-}$ secretion in seawater-acclimated rainbow trout. J. Exp. Biol. 212, 1940–1948. 10.1242/jeb.026856 19483012

[B15] Grosell M. Gilmour K. M. Perry S. F. (2007). Intestinal carbonic anhydrase, bicarbonate, and proton carriers play a role in the acclimation of rainbow trout to seawater. Am. J. Physiol. Regul. Integr. Comp. Physiol. 293, R2099–R2111. 10.1152/ajpregu.00156.2007 17761514

[B16] Grosell M. Jensen F. B. (1999). ${\text{NO}}_{2}^{-}$ uptake and ${\text{HCO}}_{3}^{-}$ excretion in the intestine of the European flounder (*Platichthys flesus*). J. Exp. Biol. 202, 2103–2110. 10393825 10.1242/jeb.202.15.2103

[B17] Heisler N. (1984). Acid-Base Regulation in Fishes. New York, NY: Academic Press.

[B18] Heuer R. M. Esbaugh A. J. Grosell M. (2012). Ocean acidification leads to counterproductive intestinal base loss in the gulf toadfish (*Opsanus beta*). Physsiol. Biochem. Zool. 85, 450–459. 10.1086/667617 22902373

[B19] Hu M. Y. Guh Y. J. Stumpp M. Lee J. R. Chen R. D. Sung P. H. (2014). Branchial ${\text{NH}}_{4}^{+}$-dependent acid-base transport mechanisms and energy metabolism of squid (*Sepioteuthis lessoniana*) affected by seawater acidification. Front. Zool. 11:55 10.1186/s12983-014-0055-z

[B20] Hu M. Y. Lee J. R. Lin L. Y. Shih T. H. Stumpp M. Lee M. F.. (2013). Development in a naturally acidified environment: Na^+^/H^+^-exchanger 3-based proton secretion leads to CO_2_ tolerance in cephalopod embryos. Front. Zool. 10:51. 10.1186/1742-9994-10-51 23988184 PMC3844404

[B21] Imsland A. K. Gunnarsson S. Foss A. Stefansson S. O. (2003). Gill Na^+^, K^+^-ATPase activity, plasma chloride and osmolality in juvenile turbot (*Scophthalmus maximus*) reared at different temperatures and salinities. Aquaculture 218, 671–683. 10.1016/S0044-8486(02)00423-4

[B22] Kajimura S. Hirano T. Moriyama S. Vakkuri O. Leppäluoto J. Grau E. G. (2004). Changes in plasma concentrations of immunoreactive ouabain in the tilapia in response to changing salinity: is ouabain a hormone in fish? Gen. Comp. Endocrinol. 135, 90–99. 10.1016/j.ygcen.2003.08.006 14644648

[B23] Kajimura S. Seale A. P. Hirano T. Cooke I. M. Grau E. G. (2005). Physiological concentrations of ouabain rapidly inhibit prolactin release from the tilapia pituitary. Gen. Comp. Endocrinol. 143, 240–250. 10.1016/j.ygcen.2005.04.002 15922343

[B24] Kiela P. R. Xu H. Gishan F. K. (2006). Apical Na^+^/H^+^ exchangers in the mammalian gastrointestinal tract. J. Physiol Pharmacol. 57(Suppl. 7), 51–79. 17228096

[B25] Kreiss C. M. Michael K. Lucassen M. Jutfeld F. Motyka R. Dupont S.. (2015). Ocean warming and acidification modulate energy budget and gill ion regulatory mechanisms in Atlantic cod (*Gadus morhua*). J. Comp. Physiol. B. 185, 767–781. 10.1007/s00360-015-0923-7 26219611 PMC4568026

[B26] Kurita Y. Nakada T. Kato A. Doi H. Mistry A. C. Chang M.-H.. (2008). Identification of intestinal bicarbonate transporters involved in formation of carbonate precipitates to stimulate water absorption in marine teleost fish. Am. J. Physiol. Regul. Integr. Comp. Physiol. 294, R1402–R1412. 10.1152/ajpregu.00759.2007 18216137

[B27] Lämmli U. K. (1970). Cleavage of structural proteins during the assembly of the head of Bacteriophage T4. Nature 227, 680–685. 5432063 10.1038/227680a0

[B28] Lannig G. Bock C. Sartoris F. J. Pörtner H. O. (2004). Oxygen limitation of thermal tolerance in cod, *Gadus morhua* L., studied by magnetic resonance imaging and on-line venous oxygen monitoring. Am. J. Physiol. Regul. Integr. Comp. Physiol. 287, R902–R910. 10.1152/ajpregu.00700.2003 15205188

[B29] Link J. S. Garrison L. P. (2002). Trophic ecology of Atlantic cod *Gadus morhua* on the northeast U.S. continental shelf. Mar. Ecol. Prog. Ser. 227, 109–123. 10.3354/meps227109

[B30] Melzner F. Göbel S. Langenbuch M. Gutowska M. A. Pörtner H. O. Lucassen M. (2009). Swimming performance in atlantic cod (*Gadus morhua*) following long-term (4-12 month) acclimation to elevated seawater pCO_2_. Aqua Toxicol. 92, 30–37. 10.1016/j.aquatox.2008.12.01 19223084

[B31] Metz J. R. van den Burg E. H. Bonga S. E. Flik G. (2003). Regulation of branchial Na(+)/K(+)-ATPase in common carp *Cyprinus carpio* L. acclimated to different temperatures. J. Exp. Biol. 206, 2273–2280. 10.1242/jeb.00421 12771175

[B32] Metzger R. Sartoris F. Langenbuch M. Pörtner H. O. (2007). Influence of elevated CO_2_ concentrations on thermal tolerance of the edible crab Cancer pagurus. J. Therm. Biol. 32, 144–151. 10.1016/j.jtherbio.2007.01.010

[B33] Michael K. Koschnick N. Pörtner H.-O. Lucassen M. (2016a). Response of branchial Na^+^/K^+^ ATPase to changes in ambient temperature in Atlantic cod (*Gadus morhua*) and whiting (*Merlangius merlangus*). J. Comp. Physiol. B. 186, 461–470. 10.1007/s00360-016-0970-8 26922791

[B34] Michael K. Kreiss C. M. Hu M. Y. Koschnick N. Bickmeyer U. Dupont S.. (2016b). Adjustments of molecular key components of branchial ion and pH regulation in Atlantic cod (*Gadus morhua*) in response to ocean acidification and warming. Comp. Biochem. Physiol. B. 193, 33–46. 10.1016/j.cbpb.2015.12.006 26688541

[B35] Munday P. L. Dixson D. L. Donelson J. M. Jones G. P. Pratchett M. S. Devitsina G. V.. (2009). Ocean acidification impairs olfactory discrimination and homing ability of marine fish. Proc. Natl. Acad. Sci. U.S.A. 106, 1848–1852. 10.1073/pnas.0809996106 19188596 PMC2644126

[B36] Nilsson G. E. Dixson D. L. Paolo D. McCormick M. I. Sorensen C. Watson S. A. (2012). Near-future carbon dioxide levels alter fish behaviour by interfering with neurotransmitter function. Nat. Cimate Change 2, 201–204. 10.1038/nclimate1352

[B37] Pastor-Soler N. M. Hallows K. R. Smolak C. Gong F. Brown D. Breton S. (2007). Alkaline pH- and cAMP-induced V-ATPase membrane accumulation is mediated by protein kinase A in epididymal clear cells. Am. J. Physiol. Regul. Cell. Physiol. 294, C488–C494. 10.1152/ajpcell.00537.2007 18160485 PMC4303256

[B38] Perry S. F. Braun M. H. Genz J. Vulesevic B. Taylor J. Grosell M.. (2010). Acid-base regulation in the plainfin midshipman (*Porichthys notatus*): an aglomerular marine teleost. J. Comp. Physiol. B. 180, 1213–1225. 10.1007/s00360-010-0492-8 20571812

[B39] Pörtner H. O. (2001). Climate change and temperature-dependent biogeography: oxygen limitation of thermal tolerance in animals. Naturwissenschaften 88, 137–146. 10.1007/s001140100216 11480701

[B40] Pörtner H. O. (2002). Physiological basis of temperature-dependent biogeography: trade-offs in muscle design and performance in polar ectotherms. J. Exp. Biol. 205, 2217–2230. 12110656 10.1242/jeb.205.15.2217

[B41] Pörtner H. O. (2012). Integrating climate-related stressor effects on marine organisms: unifying principles linking molecule to ecosystem-level changes. Mar. Ecol. Prog. Ser. 470, 273–290. 10.3354/meps10123

[B42] Pörtner H. O. Berdal B. Blust R. Brix O. Colosimo A. De Wachter B. (2001). Climate induced temperature effects on growth performance, fecundity and recruitment in marine fish: developing a hypothesis for cause and effect relationships in Atlantic cod (*Gadus morhua*) and common eelpout (*Zoarces viviparus*). Cont. Shelf Res. 21, 1975–1997. 10.1016/S0278-4343(01)00038-3

[B43] Ramnanan C. J. Storey K. B. (2006). Suppression of Na^+^/K^+^-ATPase activity during estivation in the land snail *Otala lactea*. J. Exp. Biol. 209, 677–688. 10.1242/jeb.02052 16449562

[B44] Righton D. A. Andersen K. H. Neat F. Thorsteinsson V. Steingrund P. Svedäng H. (2010). Thermal niche of Atlantic cod *Gadus morhua*: limits, tolerance and optima. Mar. Ecol. Prog. Ser. 420, 1–13. 10.3354/meps08889

[B45] Sambrook E. F. Fritsch E. F. Maniatis T. (1987). Molecular Cloning: A Laboratory Manual. New York, NY: Cold Spring Harbor.

[B46] Schneider C. A. Rasband W. S. Eliceiri K. W. (2012). NIH Image to ImageJ: 25 years of image analysis. Nat. Methods 9, 671–675. 10.1038/nmeth.2089 22930834 PMC5554542

[B47] Shum W. W. Da Silva N. Belleannée C. McKee M. Brown D. Breton S. (2010). Regulation of V-ATPase recycling via a RhoA- and ROCKII-dependent pathway in epididymal clear cells. Am. J. Physiol. Regul. Cell Physiol. 301, C31–C43. 10.1152/ajpcell.00198.2010 21411727 PMC3129830

[B48] Simpson S. D. Munday P. L. Wittenrich M. L. Manassa R. Dixson D. L. Gagliano M.. (2011). Ocean acidification erodes crucial auditory behaviour in a marine fish. Biol Lett. 7, 917–920. 10.1098/RSBL.2011.0293 21632617 PMC3210647

[B49] Staurnes M. (1993). Difference between summer and winter in gill sodium-potassium-ATPase activity and hypoosmoregulatory ability of sea farmed anadromous Arctic char. Comp. Biochem. Physiol. 105, 475–477.

[B50] Taylor J. R. Mager E. M. Grosell M. (2010). Basolateral NBCe1 plays a rate-limiting role in transepithelial intestinal ${\text{HCO}}_{3}^{-}$ secretion serving marine fish osmoregulation. J. Exp. Biol. 213, 459–468. 10.1242/jeb.029363 20086131

[B51] Tresguerres M. Levin L. R. Buck J. (2011). Intracellular cAMP signaling by soluble adenylyl cyclase. Kidney Int. 79, 1277–1288. 10.1038/ki.2011.95 21490586 PMC3105178

[B52] Tresguerres M. Levin L. R. Buck J. Grosell M. (2010). Modulation of NaCl absorption by [HCO3−] in the marine teleost intestine is mediated by soluble adenylyl cyclase. Am. J. Physiol. Regul. Integr. Comp. Physiol. 299, 62–71. 10.1152/ajpregu.00761.2009 PMC2904142 20410468

[B53] Tseng Y. C. Hu M. Y. Lin L. Y. Melzner F. Hwang P. P. (2013). CO2-driven seawater acidification differentially affects developemnt and molecular plasticity along life history of fish (*Oryzias latipes*). Comp. Biochem. Physiol. 165, 119–130. 10.1016/j.cbpa.2013.02.005 23416137

[B54] Tseng Y. C. Liu S. T. Hu M. Y. Chen R. D. Lee J. R. Hwang P. P. (2014). Brain functioning under acute hypothermic stress supported by dynamic monocarboxylate utilization and transport in ectothermic fish. Front. Zool. 11:53 10.1186/s12983-014-0053-1

[B55] Walther K. Sartoris F. Bock C. Pörtner H. O. (2009). Impact of anthropogenic ocean acidification on thermal tolerance of the spider crab *Hyas araneus*. Biogeosci. Discuss 6, 2837–2861. 10.5194/bg-6-2207-2009

[B56] Wilson R. W. Gilmour K. M. Henry R. P. Wood C. (1996). Intestinal base excretion in the seawater-adapted rainbow trout: a role in acid-base balance? J. Exp. Biol. 199, 2331–2343. 9320250 10.1242/jeb.199.10.2331

[B57] Wilson R. W. Millero F. J. Taylor J. R. Walsh P. J. Christensen V. Jennings S.. (2009). Contribution of fish to the marine inorganic carbon cycle. Science 323, 359–362. 10.1126/science.1157972 19150840

