# Supplementary material for: Temperature Modulates the Effects of Ocean Acidification on Intestinal Ion Transport in Atlantic Cod, Gadus morhua
Source: Front Physiol. 2016 Jun 2;7:198. doi: 10.3389/fphys.2016.00198 (PMC4889603; doi:10.3389/fphys.2016.00198)
Supplement: Supplementary file 1 [file Table1.PDF]

Gene	10℃			18℃		
	pH 8.1	pH 7.8	pH 7.6	pH 8.1	pH 7.8	pH 7.6
CA2a	70242.13±7879.17	74939.84±5456.01	128140.67±16147.09	865863.57±301026.69	0.78±0.19	1115.82±665.24
CA2b	1.90±0.48	2.83±0.80	2.38±0.83	4.43±0.93	1.87±0.38	4.93±1.20
CA4a	Non-Detectable			Non-Detectable		
CA4b	0.94±0.24	1.12±0.23	3.31±0.62	1.16±0.16	1.12±0.31	5.56±2.49
CA4c	Non-Detectable			Non-Detectable		
CA15a	0.97±0.15	1.09±0.15	8.79±1.04	0.64±0.12	0.69±0.11	0.62±0.28
CA15b	Non-Detectable			Non-Detectable		
CA15c	Non-Detectable			Non-Detectable		
VHAa	1.11±0.18	1.63±0.26	1.38±0.14	1.34±0.22	1.23±0.20	1.78±0.40
VHAb	Non-Detectable			Non-Detectable		
NHE1a	1.18±0.24	0.88±0.15	1.22±0.34	0.94±0.12	0.78±0.10	0.74±0.12
NHE1b	Non-Detectable			Non-Detectable		
NHE2	Non-Detectable			Non-Detectable		
NHE3	1.61±0.43	1.27±0.25	4.17±0.74	1.32±0.27	0.83±0.31	0.85±0.25
ATP1A1	0.98±0.12	1.53±0.11	1.61±0.13	1.06±0.11	0.92±0.19	0.83±0.23
Rhag	Non-Detectable			Non-Detectable		
Rhbg	1.57±0.36	30.99±6.65	30.91±5.83	71.05±16.64	30185.42±6277	17915574±8428697.3
Rhcg1	Non-Detectable			Non-Detectable		
Rhcg2	Non-Detectable			Non-Detectable		
AE1a	Non-Detectable			Non-Detectable		
AE1b	Non-Detectable			Non-Detectable		
NBCa	1.11±0.17	1.59±0.09	4.78±0.95	2.85±0.51	3.81±0.69	3.94±0.82
NBCb	0.89±0.15	0.68±0.08	0.63±0.13	0.95±0.18	1.10±0.22	0.99±0.16
SLC26A3.1	Non-Detectable			Non-Detectable		
SLC26A3.2	0.97±0.22	2.88±0.53	3.12±0.53	2.87±0.75	5.14±1.19	1.04±0.23
SLC26A5	Non-Detectable			Non-Detectable		
SLC26A6a	0.87±0.09	0.35±0.04	5.46±1.67	3.32±1.06	1.01±0.30	2.81±1.35
SLC26A6b	Non-Detectable			Non-Detectable		
SLC26A6c	Non-Detectable			Non-Detectable		
